# Oncogenic Activation of YAP Signaling Sensitizes Ferroptosis of Hepatocellular Carcinoma *via* ALOXE3-Mediated Lipid Peroxidation Accumulation

**DOI:** 10.3389/fcell.2021.751593

**Published:** 2021-12-16

**Authors:** Yifei Qin, Zhuo Pei, Zhuan Feng, Peng Lin, Shijie Wang, Yong Li, Fei Huo, Quancheng Wang, Zhiping Wang, Zhi-Nan Chen, Jiao Wu, Yi-Fei Wang

**Affiliations:** ^1^ Guangdong Provincial Engineering Center of Topical Precise Drug Delivery System, School of Pharmacy, Guangdong Pharmaceutical University, Guangzhou, China; ^2^ National Translational Science Center for Molecular Medicine, Department of Cell Biology, Fourth Military Medical University, Xi’an, China; ^3^ Guangzhou (Jinan) Biomedical Research and Development Center, Guangzhou, China

**Keywords:** ferroptosis, YAP, ALOXE3, sorafenib, hepatocellular carcinoma, lipid peroxidation

## Abstract

Ferroptosis, a form of programmed cell death process driven by iron-dependent lipid peroxidation, plays an important role in tumor suppression. Although previous study showed that intracellular Merlin-Hippo signaling suppresses ferroptosis of epithelial tumor cells through the inactivation of YAP signaling, it remains elusive if the proto-oncogenic transcriptional co-activator YAP could serve as a potential biomarker to predict cancer cell response to ferroptosis-inducing therapies. In this study, we show that both total YAP staining and nuclear YAP staining were more prevalent in HCC tissues than in nontumorous regions. Compared to low-density HCC cells, high-density cells showed decreased nuclear localization of YAP and conferred significant resistance to ferroptosis. Oncogenic activation of YAP signaling by overexpression of YAP(S127A) mutant sensitized ferroptosis of HCC cells cultured in confluent density or in the 3D tumor spheroid model. Furthermore, we validated the lipoxygenase ALOXE3 as a YAP-TEAD target gene that contributed to YAP-promoted ferroptosis. Overexpression of ALOXE3 effectively increased the vulnerability of HCC cells to ferroptotic cell death. In an orthotopic mouse model of HCC, genetic activation of YAP rendered HCC cells more susceptible to ferroptosis. Finally, an overall survival assay further revealed that both a high expression of YAP and a low expression of GPX4 were correlated with increased survival of HCC patients with sorafenib treatment, which had been proven to be an inducer for ferroptosis by inhibition of the xc-amino acid antiporter. Together, this study unveils the critical role of intracellular YAP signaling in dictating ferroptotic cell death; it also suggests that pathogenic alterations of YAP signaling can serve as biomarkers to predict cancer cell responsiveness to future ferroptosis-inducing therapies.

## Introduction

Programmed cell death (PCD) plays important roles in normal biology, and its dysregulation impacts human disease (Green and Llambi 2015). Apoptosis has long been the classical example of PCD, but in recent years the field has rapidly grown to include other forms of PCD such as necroptosis and pyroptosis (Linkermann and Green 2014; Conrad, Angeli et al., 2016; Galluzzi, Vitale et al., 2018). Ferroptosis is yet another newly emerged PCD modality (Yang and Stockwell 2016; Jiang, Stockwell et al., 2021). Ferroptosis is triggered by an imbalance of cellular redox coupled with strong cellular metabolism, leading to a wave of iron-dependent cellular lipid peroxidation and, ultimately, cell death (Yang and Stockwell 2016). In addition to Fenton reaction, which provides hydroxyl radicals that subtract hydrogen (H) from lipid to form a lipid radical (L•) as the start of the non-enzymatic reaction of lipid peroxidation, the levels of lipid peroxides can be enhanced enzymatically by the lipoxygenase family (including ALOXE3, ALOX5, ALOX12, ALOX12B, ALOX15 and ALOX15B in mammalian cells) (Chu, Kon et al., 2019).

The role of ferroptosis in various diseases has been demonstrated. In multiple experimental models, inhibition of ferroptosis attenuated neurodegeneration and ischemia/reperfusion-induced organ damage (Friedmann Angeli, Schneider et al., 2014; Linkermann, Skouta et al., 2014; Gao, Monian et al., 2015; Guiney, Adlard et al., 2017). Furthermore, a prominent role for ferroptosis in cancer is also emerging. For example, ferroptosis contributes to the tumor suppressive function of p53, due to the ability of p53 to suppress and stimulate the transcription of SLC7A11 (a subunit of cystine transporter, which suppresses ferroptosis) and glutaminase-2 (which supports ferroptosis via glutaminolysis), respectively (Jiang, Kon et al., 2015; Jennis, Kung et al., 2016). Strikingly, it was demonstrated that numerous types of therapy-resistant cancer cells, especially those with mesenchymal and de-differentiated characteristics, are more susceptible to ferroptosis (Hangauer, Viswanathan et al., 2017; Viswanathan, Ryan et al., 2017; Tsoi, Robert et al., 2018). Therefore, the induction of ferroptosis might be a promising therapeutic approach for killing such otherwise therapy-resistant, mesenchymal cancer cells with high metastatic potential.

Intercellular cadherin clustering can signal through the Merlin tumor suppressor to activate intracellular Hippo signaling (Kim, Koh et al., 2011; Meng, Moroishi et al., 2016), which in turn catalyzes the phosphorylation of YAP at serine 127 (Zhao, Wei et al., 2007) by Lats1/2. YAP is an oncogenic transcriptional co-activator; it forms a protein complex with various transcription factors, including members of the TEAD family of transcription factors, to modulate gene transcription (Choi, Zhang et al., 2015; Stein, Bardet et al., 2015; Panciera, Azzolin et al., 2017). In its phosphorylated state, YAP is sequestered to the cytoplasm and thus cannot exert its activity as a transcriptional co-activator (Zhao, Wei et al., 2007; Li, Cooper et al., 2012; Varelas and Wrana 2012). In mesothelioma cells, antagonizing the E-cadherin-Merlin-Hippo signaling axis unleashes the activity of the proto-oncogenic transcriptional co-activator YAP to promote ferroptosis through upregulation of multiple ferroptosis modulators, including acyl-CoA synthetase long chain family member 4 (ACSL4) and transferrin receptor (Wu, Minikes et al., 2019). Overexpression of YAP also sensitizes cancer cells to ferroptosis via regulating the E3 Ligase S-phase kinase-associated protein 2 (SKP2) (Yang, Lin et al., 2021). However, the mechanisms underlying the sensitivity of YAP-activated cancer cells to ferroptosis are not completely defined. It also remains unclear if YAP signaling could predict the responsiveness of cancer cell to ferroptosis-inducing therapies.

In this study, by using models of monolayer cell culture, 3-dimensional (3D) tumor spheroid and tumor xenograft, we demonstrate that YAP is able to promote ferroptosis and lipid peroxides accumulation by transcriptionally upregulating the lipoxygenase ALOXE3. As the pathogenic alterations of oncogene YAP culminate in the development of a broad range of human tumor types, including HCC, this finding suggests that YAP-ALOXE3 signaling may be useful as a biomarker to predict HCC cell responsiveness to ferroptosis induction. Indeed, in the xenograft mouse model we demonstrate that the activation of YAP renders tumor cells more sensitive to ferroptosis induced by sorafenib, which induces ferroptosis by inhibition of the xc-amino acid antiporter.

## Materials and Methods

### Cell Culture

LM3 liver cancer cell line was obtained from BeNa culture collection (BNCC102270), HuH-7, SK-HEP-1, HepG2, Hepa1-6 liver cancer cell lines, and the HEK-293T cell line were obtained from the American type culture collection (ATCC, United States) and have been proven to be negative for *mycoplasma* contamination. All cells were incubated in a 37°C incubator with 5% CO2 and cultured in Dulbecoo’s modified Eagle’s medium (DMEM) supplemented with 10% fetal bovine serum (FBS). YAP^S127A^-LM3, YAP^S127A^-Hepa1-6, shNT-LM3/SK-HEP-1, shYAP-LM3/SK-HEP-1 cells were generated by retroviral or lentiviral transfection.

### SiRNA Transfection

The design of siRNA interference target and the preparation of double-stranded siRNA targeting ALOXE3 were completed by GenePharma (Shanghai, China). siALOXE3#1: sense, CCUGCUAUCAGUGGAUUGATT, antisense, UCAAUCCACUGAUAGCAGGTT. siALOXE3#2: sense, GCCCAAUGUUCGAUACUCATT, antisense, UGAGUAUCGAACAUUGGGCTT. siALOXE3#3: sense, GUACCUGAAUGGUGUCAAUTT, antisense, AUUGACACCAUUCAGGUACTT.Parental LM3 and YAP^S127A^ - LM3 cells in the logarithmic growth phase were routinely digested for cell counting. Cells prepared at 3.0 × 10^5^ cells/well were seeded in a six-well plate. They were cultured to cell confluence of 60–70% prior to transfection. After 24 h of transfection, 200 µL serum-free Opti-MEM plus 7.5 µL PEI, and 200 µL serum-free Opti-MEM plus 5 μg siRNA were mixed and incubated at room temperature for 5 min, and then the mixture was incubated together for 20 min. Transfection mixture was applied to each well with 2 ml of serum-free medium replaced. After transfection at 37°C for 6 h, DMEM containing 10% FBS was replaced. Transfected cells at 24 and 72 h were respectively used for qRT-PCR and Western blotting. Transfected cells at 24 h were re-seeded and treated with RSL3 to measure cell death.

### Plasmids, Retroviral and Lentiviral Infection

The 8xGTIIC-luciferase reporter was from the Piccolo laboratory (Addgene plasmid 34615). pQCXIH-Flag-YAP-S127A was from the Guan laboratory (Addgene plasmid 33092). SFB-ALOXE3 was a gift from the Chu laboratory (Shandong University). Lentiviral vectors encoding shRNAs targeting human YAP were gifts from the Jiang laboratory (Memorial Sloan Kettering Cancer Center). For retroviral particle assembly, retroviral vector pQCXIH-Flag-YAP-S127A was co-transfected with gag/pol (Addgene) and VSV-G (Addgene) into 293T cells using PEI. Lentiviruses were produced by the co-transfection of the lentiviral vector with the Delta-VPR envelope and CMV VSV-G packaging plasmids into HEK-293T cells using PEI (Sigma-Aldrich). The progeny viruses released from 293T cells were filtered and collected 48 h after transfection, and used to infect HCC cells.

### Western Blotting

Briefly, cells were scraped into ice-cold radioimmunoprecipitation assay (RIPA) buffer and 1% Halt protease and phosphatase inhibitor cocktail. Briefly, cells were scraped into ice-cold radioimmunoprecipitation assay (RIPA) buffer and 1% Halt protease and phosphatase inhibitor cocktail. Cell lysates were centrifuged at 14,000 rpm for 15 min at 4°C, and supernatants were collected. Protein concentrations were determined using the Bradford method. Cellular proteins were separated by 10% SDS-PAGE, transferred onto PVDF membranes (0.45 μM, IPVH00010, Millipore), and blocked with 5% skim milk at room temperature for 1 h. Membranes were incubated with the following primary antibodies: anti-YAP1 Polyclonal Antibody (PA5-87568, Invitrogen, 1:2000), anti-ALOXE3 (ab118470, Abcam, 1:1000), anti-β-actin (ab8226, Abcam, 1:3000) and anti-GAPDH (60004-1-Ig, Proteintech, 1:5000) overnight at 4°C. They were washed and incubated with the horseradish peroxidase conjugated anti-rabbit or mouse IgG (H + L) (1:3000; Proteintech). The enhanced chemiluminescence solution (GE Healthcare) was used for visualizing protein expression.

### Cell Death and Cell Viability

Four HCC cell lines (SK-HEP-1, HuH-7, LM3, HepG2) were seeded in 96-well plates with high-density group (3 × 10^4^ cells/well) and low-density group (3 × 10^3^ cells/well). RSL3 (Sigma) was diluted to 4 μM, 2 μM, 1 μM, 0.5 μM, and 0.25 μM respectively. Ferrostatin-1 (MCE) was used as a specific ferroptosis inhibitor. Cells were collected for SYTOX Green (Invitrogen) and Hoechst 33342 (Beyotime) staining for analyzing cell death by microscopy and the number of dead cells was counted using ImageJ software.

Cell viability was measured by CellTiter-Glo luminescent cell viability assay (Promega). Briefly, 100 μL of assay reagent was added to each well and mixed at room temperature. After 15 min, intracellular ATP content was measured by a luminescence multilabel counter.

### CCK-8 Assay

The ability of cell proliferation was assessed using CCK-8 (Catalog No. C0005, TargetMoI) according to the manufacturer’s instructions. Parental LM3 cells, ShYAP LM3 cells, Parental SK-HEP-1 cells and ShYAP SK-HEP-1 cells were seeded in 96-well plates (3 × 10^3^ cells/well) and cultured overnight, followed by treatment with RSL3 for 24 h. 90 μL culture medium plus 10 μL CCK-8 solution was added to each well and then incubated about 1–4 h at 37°C until the color of medium turned orange. The absorbance of each well was measured by a microplate reader at an excitation wavelength of 450 nm and then the cell activity was calculated.

### Lipid Peroxidation Assay

For analyzing RSL3-induced cell lipid ROS, five HCC cell lines (SK-HEP-1, HuH-7, LM3, HepG2, Hepa1-6) were seeded in 6-well plates divided into high-density group (3 × 10^5^ cells/well) and low-density group (3 × 10^4^ cells/well) and cultured overnight, and then Fer-1 and/or RSL3 was added to the well plates. After 6 h cell induction, a ratiometric lipid peroxidation sensor (lipid peroxidation assay kit, ab243377 Abcam) was added to cells and incubated for 30 min. Then, cells were collected, resuspended in 250 μL of serum-free medium and examined by flow cytometry with FITC and PE channels [Ex = 488 nm and Em = 530 nm (FITC) or 572 nm (PE)] within 2 h of staining. The sensor in the lipid peroxidation kit changes its fluorescence from PE to FITC upon peroxidation by lipid ROS in cells, thus enabling ratio metric measurement of lipid peroxidation (the decreasing PE/FITC ratio means increasing lipid peroxidation).

### Immunofluorescence (IF)

For immunofluorescence (IF) staining, HCC cells were seeded at 3.0 × 10^5^ cells or 3.0 × 10^4^ cells per 35 mm confocal dish. After overnight cell culture, cells were fixed in 4% PFA for 15 min, and washed with PBS. They were blocked with 5% goat serum in PBS and then incubated at 4°C overnight with the anti-YAP (rabbit, PA5-87568, Invitrogen, 1:200). On the next day, cells were washed 3 times with PBS, and incubated with the Alexa Fluor 488 anti-rabbit IgG (Invitrogen, 1:200) at room temperature for 1 h. Nuclei were stained with 4,6-diamidino-2-phenylindole (DAPI, Beyotime). IF images were acquired using a confocal microscope (Nikon).

### Immunohistochemistry (IHC)

Immunohistochemistry was carried out to explore positive expressions of YAP1, ALOXE3, PTGS2, GPX4, Ki67 and 4HNE in human HCC specimens and xenografts generated from HCC cells. The high-throughput tissue microarray was purchased from Shaanxi Avila Biotechnology Co., Ltd. (Cat. No. DC-liv11014). Of the 208 cases involved in the commercial tissue microarray, 16 cases are normal liver tissue, and 180 cases are hepatocellular carcinoma or mixed hepatocellular carcinoma. Of the 180 cases of hepatocellular carcinoma, 6 sections fell off the slides or showed large area of necrosis, so 174 cases were retained for further analysis. Some cases lack the information about grades, which were excluded for analysis about grades. The thickness of tissue sections is 5 μm. Antigen retrieval was performed in citrate buffer in 100°C water bath for 2 min, aiming to eliminate endogenous peroxidase activity, and 5% BSA was added to block nonspecific epitopes. Sections were incubated with primary antibodies as follows: Anti-YAP1 (1:200; Invitrogen), anti-4HNE (1:200; Invitrogen), anti-ALOXE3 (1:400; Abcam), anti-PTGS2 (1:200; Invitrogen), anti-Ki67 (1:250; Invitrogen) and anti-GPX4 (1:250; Invitrogen) with a standard avidin-biotin HRP detection system according to manufacturer’s instructions (anti-mouse/rabbit HRP-DAB Cell & Tissue Staining Kit, R&D Systems). Tissues were counterstained with haematoxylin, dehydrated and mounted. In all cases, antigen retrieval was done with the BD Retrievagen (pH 6.0) Antigen Retrieval Systems. Slices were baked for 2 h, and the remaining steps were the same as the IHC steps. Primary antibodies of anti-YAP1 (1:200; Invitrogen) and anti-ALOXE3 (1:400; Abcam) were used. The product of the staining intensity score A and the staining positive cell ratio score B were considered as the scoring standard. Briefly, scoring standard of A: 0, negative staining; 1, weak intensity; 2, medium intensity; 3, high intensity. Scoring standard of B: 0, negative staining; 1, positive cell number ≤25%; 2, 25% < positive cell number <50%; 3, 50% < positive cell number <75%; 4, positive cell number ≥75%. The final score is obtained by multiplying A and B for analyzing.

### RNA-Sequencing and Gene Expression Analysis

Total RNA was extracted from YAP^S127A^ - LM3 cells and parental LM3 cells using TRIzol (Invitrogen). The quality of RNA was assessed with an Agilent 2,100 Bioanalyzer (Agilent Technologies, Palo Alto, CA, United States) and checked with RNase free agarose gel electrophoresis. Eukaryotic mRNA was enriched by Oligo (dT) beads, while prokaryotic mRNA was enriched by removing rRNA by Ribo-ZeroTM Magnetic Kit (Epicentre, Madison, WI, United States). After enrichment, mRNA was fragmented using fragmentation buffer and reverse transcribed into cDNA with random primers. Second-strand cDNA were synthesized by DNA polymerase I, RNase H, dNTP and buffer, and the cDNA fragments were purified with the QiaQuick PCR extraction kit (Qiagen, Venlo, Netherlands), end repaired, poly (A) added, and ligated to Illumina sequencing adapters. The ligation products were size selected by agarose gel electrophoresis, PCR amplified, and sequenced using Illumina Novaseq6000 by Gene Denovo Biotechnology Co. (Guangzhou, China). For GSEA, the normalized enrichment score (NES) was used to quantify enrichment magnitude and statistical significance, respectively. The RNA-seq data generated in this study have been deposited in the NCBI Sequence Read Archive (SRA) database under the accession code PRJNA762931 [https://www.ncbi.nlm.nih.gov/bioproject/PRJNA762931].

### *In vivo* Xenograft Mouse Model

Female nude mice with 4 weeks old were injected with 2 × 10^6^ Parental LM3 or YAP^S127A^LM3 cells subcutaneously. Visible tumors appeared after 1 week. Tumor size was measured by an electronic caliper every 2 days and calculated using the formula: Tumor size (mm^3^) = 0.5 × length × width^2^. After another 1 week, mice were randomly separated into 4 group of 10 mice per cage with roughly tumor size (100 mm^3^). They were treated with saline or 50 mg/kg Sorafenib (Selleck) dissolved in 5% DMSO+5% Tween-80 + 40% PEG-300 + 50% ddH_2_O *via* gavage once a day. At 12th week, mice were sacrificed by cervical dislocation, and subcutaneous tumors were dissected, photographed and weighed. The tumors were appropriately segmented, and one part was immediately frozen in liquid nitrogen and stored at −80°C, and the remaining were soaked in 10% formalin, followed by sectioning and immunohistochemical staining.

### Dual-Luciferase Reporter Assay

The transcriptional activity of YAP with its primary binding partners, the TEAD family of transcription factors was monitored by an 8xGTIIC-luciferase reporter assay. The 8xGTIIC-luciferase reporter was from the Piccolo laboratory (Addgene plasmid 34615). Parental LM3 or YAP^S127A^-LM3 cells were plated in 12-well plates at 1.5 × 10^5^ cells per well and incubated overnight. Cells were transfected the next day with 20 ng of 8xGTIIC-luciferase reporter and 1 ng of Renilla luciferase reporter plasmid (pRL-CMV; Promega). The transcriptional regulation of ALOXE3 was also investigated using the dual-luciferase reporter assay system. To analyze the regulation of ALOXE3 by TEAD4, we co-transfected pCMV6-Entry-TEAD4 with pGL3-ALOXE3 (−3000/+250) and pRL-CMV into HEK293 cells. At 24 h post-transfection, luciferase activities were assayed using the dual-luciferase reporter assay system (Promega) according to the manufacturer’s instructions. Firefly and Renilla luciferase activities were quantified using a luminometer, with firefly luciferase activity normalized for transfection efficiency based on Renilla luciferase activity. To analyze the regulation of ALOXE3 by YAP activation, we contransfected pGL3-ALOXE3 and pRL-CMV into parental cells and YAP^S127A^-overexpressing cells.

### ChIP

A ChIP assay was carried out using a ChIP Kit (ab500, Abcam). Briefly, cell lysates were incubated with beads and anti-TEAD4 (ab58310) antibody. DNA fragments were assessed by quantitative PCR with reverse transcription (qRT–PCR) using the primer sequences. Quantitative PCR reactions generated products from the promoter region of the ALOXE3 gene. Primers were designed based on TEAD4-binding peak regions depicted in the ENCODE TEAD4 ChIP–seq datasets. The following primers were used: ALOXE3-1: forward 5′-GGC​GAA​ATC​CCG​TCT​CTA​CA-3′, and reverse 5′-CTC​TCC​TGC​CTC​AGC​CTC​CT-3'; ALOXE3-2: forward 5′-CAC​CAC​AAC​CTC​TGC​CTC​CT-3′, and reverse 5′-TGG​TGA​AAC​CCC​ATC​TCT​ACT​AAA-3'; ALOXE3-3: forward 5′-GCC​TCA​GCT​TCC​CAA​GTA​GCT-3′, and reverse 5′-TGG​GCG​ACA​AGA​GCA​ACA​CT-3'; ALOXE3-4: forward 5′-GGT​GGC​GCG​ATC​TTA​GCT​C-3′, and reverse 5′-TGG​CGG​GTG​CCT​GTA​GTC-3'.

### Generation of 3D Spheroids

HCC cells (SK-HEP-1, HuH-7, LM3, HepG2) and YAP^S127A^LM3 cells were prepared to suspension at 2 × 10^4^ cells/mL. After centrifugation at 600 *g* for 5 min and mixture in 2.5% (*v*/*v*) Matrigel (Corning), cells were applied on 96-well Black/Clear Ultra-Low Attachment Microplate (22918053, Corning) and were incubated at 37°C, 5% CO_2_ for 48–72 h formation of single spheroids of cells. Spheroids were then treated with RSL3 and Fer-1 in medium containing Matrigel for the indicated time. After 90% cells dead, cell viability was assessed by CellTiter-Glo® 3D Cell Viability Assay (Promega).

### Real-Time qPCR

RNA was extracted using the TRIzol reagent (Invitrogen). cDNA was synthesized using Superscript first strand synthesis kit (Invitrogen) according to the manufacture’s protocol. RT–PCR was performed a TB Green PCR kit (TaKaRa, Otsu, Japan) on the Mxpro system to determine the expression levels of the genes. Primers used in qPCR were listed as follows: ALOXE3-Forward: 5′-CAA​GGA​CTC​TTG​GTA​CTG​TAG​C-3′, ALOXE3-Reverse: 5′-TAG​CCT​TCA​ATC​CAC​TGA​TAG​C-3’; GPX2-Forward: 5′-GGT​AGA​TTT​CAA​TAC​GTT​CCG​GG-3′, GPX2-Reverse: 5′-TGA​CAG​TTC​TCC​TGA​TGT​CCA​AA-3’; GSTk1-Forward: 5′-AGA​ACC​AGC​TCA​AGG​AGA​CC-3′, GSTk1- Reverse: 5′-CAT​CCA​CTT​CTC​TCC​CAG​CA-3’;

GSTM4-Forward: 5′-ACT​TGA​TTG​ATG​GGG​CTC​AC-3′, GSTM4- Reverse: 5′-TCT​CCA​AAA​TGT​CCA​CAC​GA-3’; SOD2-Forward: 5′-TTT​CAA​TAA​GGA​ACG​GGG​ACA​C-3′, SOD2- Reverse: 5′-GTG​CTC​CCA​CAC​ATC​AAT​CC-3’; GCLC-Forward: 5′-GGC​ACA​AGG​ACG​TTC​TCA​AGT-3′, GCLC- Reverse: 5′-CAG​ACA​GGA​CCA​ACC​GGA​C-3’; IDH1-Forward: 5′-CAC​TAC​CGC​ATG​TAC​CAG​AAA​GG-3′, IDH1-Reverse: 5′-TCT​GGT​CCA​GGC​AAA​AAT​GG-3’; GAPDH-Forward: 5′-CAG​GTG​GTC​TCC​TCT​GAC​TT-3′, GAPDH-Reverse: 5′-CCA​AAT​TCG​TTG​TCA​TAC​CA-3’.

### Statistical Analysis

All statistical analyses were performed using GraphPad Prism 8.0 Software. Data in Figure 8A are presented as mean ± SEM. Other data are presented as mean ± SD. *p* values were determined by Student’s two-tailed t-test or one-way ANOVA. *p*-values < 0.05 were considered to be statistically significant.

## Results

### Increased Expression and Nuclear Localization of YAP in Hepatocellular Carcinoma

**FIGURE 1:**
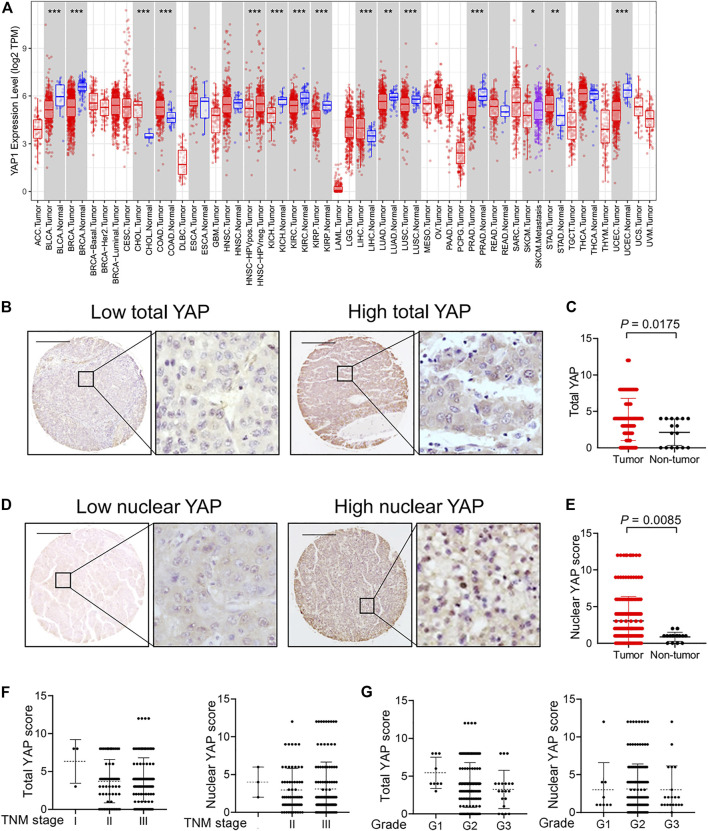
The expression and localization of YAP in human hepatocellular carcinoma tissues. **(A)** YAP expression in different cancer types via the Tumor Immune Estimation Resource (TIMER) database. Adrenocortical carcinoma (ACC), bladder urothelial carcinoma (BLCA), breast invasive carcinoma (BRCA), cervical squamous cell carcinoma (CESC), cholangiocarcinoma (CHOL), colon adenocarcinoma (COAD), lymphoid neoplasm diffuse large b-cell lymphoma (DLBC), esophageal carcinoma (ESCA), glioblastoma multiforme (GBM), head and neck squamous cell carcinoma (HNSC), kidney chromophobe (KICH), kidney renal clear cell carcinoma (KIRC), kidney renal papillary cell carcinoma (KIRP), acute myeloid leukemia (LAML), lower grade glioma (LGG), liver hepatocellular carcinoma (LIHC), lung adenocarcinoma (LUAD), lung squamous cell carcinoma (LUSC), mesothelioma (MESO), ovarian serous cystadenocarcinoma (OV), pancreatic adenocarcinoma (PAAD), pheochromocytoma and paraganglioma (PCPG), prostate adenocarcinoma (PRAD), rectum adenocarcinoma (READ), sarcoma (SARC), skin cutaneous melanoma (SKCM), stomach adenocarcinoma (STAD), testicular germ cell tumors (TGCT), thyroid carcinoma (THCA), thymoma (THYM), uterine corpus endometrial carcinoma (UCEC), uterine carcinosarcoma (UCS), uveal melanoma (UVM). **(B)** Expression of YAP protein in hepatocellular carcinoma tissues. Scale bars, 200 μm. **(C)** Expression score of YAP in HCC tissues (*n* = 174) and normal liver tissue (*n* = 16) assessed by immunohistochemistry. **(D)** The localization of YAP in nucleus in hepatocellular carcinoma tissues. **(E)** The score of nuclear YAP expression in HCC tissues (*n* = 174) and normal liver tissues (*n* = 16). **(F)** YAP expression and nuclear YAP expression in tumor tissues of HCC patients with different TNM classification. I, *n* = 3 patients. II, *n* = 61 patients. III, *n* = 110 patients. **(G)** YAP expression and nuclear YAP expression in tumor tissues of HCC patients with different grades. G1, *n* = 9 patients. G2, *n* = 140 patients. G3, *n* = 20 patients.

The expression of YAP was investigated by Tumor Immune Estimation Resource (TIMER). We found that YAP was highly expressed in the tumor tissues of cholangiocarcinoma (CHOL), colon adenocarcinoma (COAD), liver hepatocellular carcinoma (LIHC) and stomach adenocarcinoma (STAD) (Figure 1A). We performed immunohistochemistry to investigate the expression and localization of YAP in human HCC tissues and normal tissues using a high-throughput tissue microarray analysis. In HCC tissues, the total YAP staining was enhanced (Figures 1B,C), and YAP nuclear staining was also more prevalent than in nontumorous regions (Figures 1D,E). However, there was no significant correlation between total YAP or nuclear YAP level and Tumor-Node-Metastasis (TNM) stages (Figure 1F) or grades of HCC (Figure 1G).

### Cell Confluence Dictates Ferroptosis Sensitivity in Cultured HCC Cells

**FIGURE 2:**
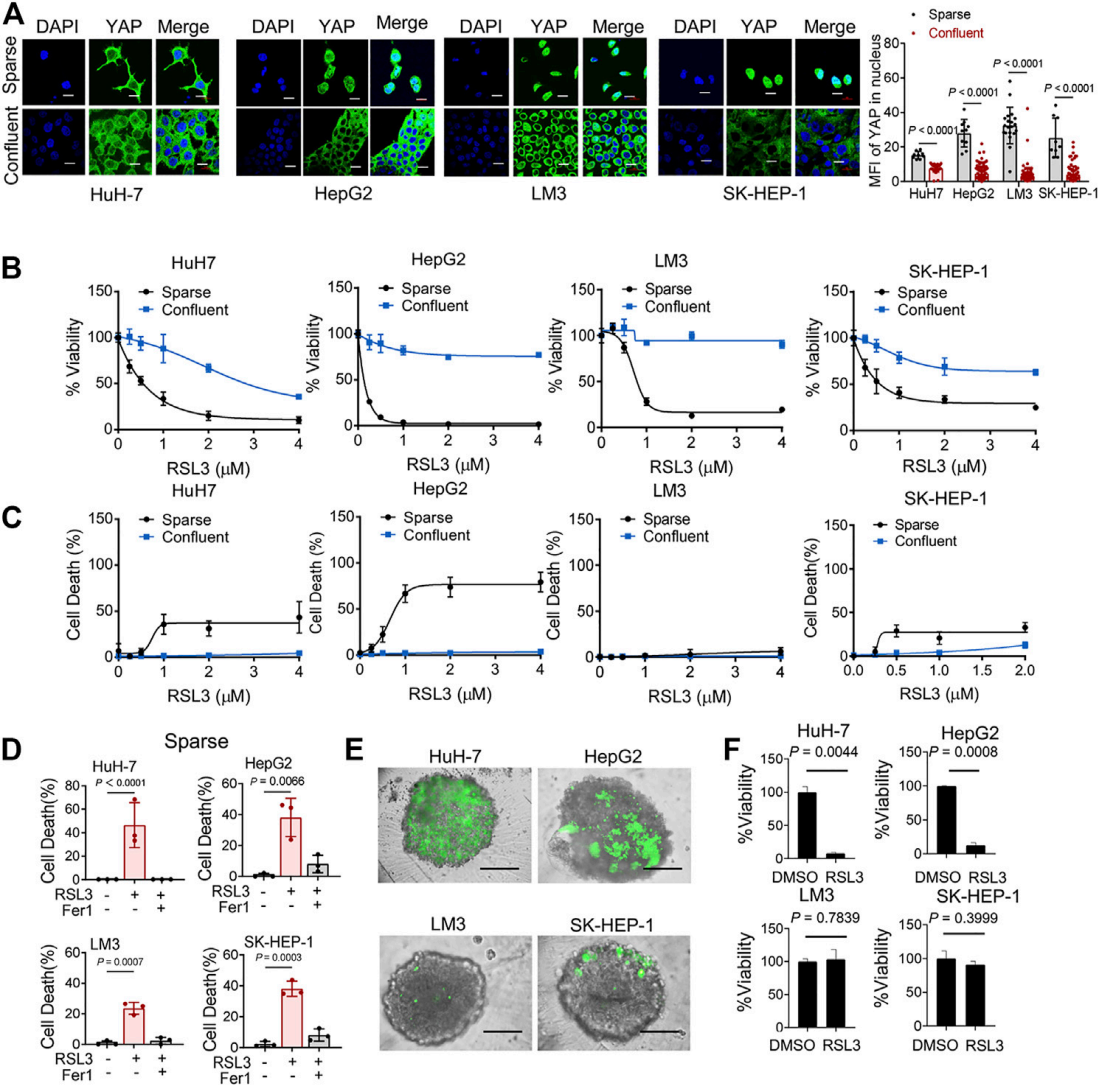
Cell Confluence-dependent regulation of ferroptosis in cultured human HCC cell lines. **(A)** HuH-7, HepG2, LM3 and SK-HEP-1 HCC cells were cultured at different cell densities. Left, YAP localization was assessed by immunofluorescence. Sparse, 3 × 10^4^/35 mm confocal dish. Confluent, 3 × 10^5^/35 mm confocal dish. Scale bars, 20 μm. Right, the expression of YAP in nucleus was quantified by ImageJ. HuH-7, sparse *n* = 9 cells from 3 images, confluent *n* = 46 cells from 3 images. HepG2, sparse *n* = 10 cells from 3 images, confluent *n* = 100 cells from 3 images. LM3, sparse n = 20 cells from 3 images, confluent *n* = 99 cells from 3 images. SK-HEP-1, sparse *n* = 9 cells from 3 images, confluent *n* = 85 cells from 3 images. **(B)** HCC cells were cultured under sparse or confluent conditions. Ferroptosis was induced by 18 h treatment of RSL3 with indicated concentrations. Cell viability was assayed by measuring cellular ATP levels. *n* = 3 biologically independent samples per condition. **(C)** Cell death was measured by SYTOX green staining following 12 h treatment of RSL3 with indicated concentrations. *n* = 3 biologically independent samples per condition. **(D)** Sparse HCC cells were treated with or without RSL3 (1 μM) or ferroptosis inhibitor Ferrostatin-1 (Fer-1, 1 μM) as indicated for 15 h. Cell death was measured. *n* = 3 biologically independent samples per condition. **(E,F)** Three-dimensional spheroids were treated with 2 μM RSL3 for 20 h. Dead cells were stained by SYTOX green **(E)** and cell viability was assayed by measuring cellular ATP **(F)**. Scale bars, 200 μm *n* = 2 biologically independent samples per condition.

Intercellular interaction of epithelial cells can signal to intracellular Hippo pathway (Kim, Koh et al., 2011). The Hippo pathway involves multiple players, such as the tumor suppressor Merlin and a kinase cascade comprising Mst1/2 and Lats1/2. Lats1/2 suppresses the function of the pro-oncogenic transcriptional cofactor YAP by inducing its nuclear exclusion through phosphorylation at its S127 residue. We examined the localization of YAP in 4 HCC cell lines. As expected, as HCC cells grew more confluent, decreased nuclear localization of YAP were observed (Figure 2A). Previous study has confirmed the role of YAP in promoting ferroptosis in mesothelioma cells, and also suggested that the transcriptional regulation mediated by YAP-TEAD interaction is responsible for this effect (Wu, Minikes et al., 2019). To test this possibility in HCC cells, we seeded different numbers of HCC cells in culture dishes. We found that higher cell confluence conferred significant resistance to ferroptosis induced by a covalent inhibitor of GPX4, RSL3, which is commonly used as an inducer for ferroptosis (Stockwell, Friedmann Angeli et al., 2017) (Figures 2B,C). Ferrostatin-1 (Fer-1), a selective inhibitor of ferroptosis, significantly blocked cell death of sparse cells (Figure 2D). Among the HCC cell lines tested, high-density LM3 cells showed the strongest resistance at high cell density to ferroptosis induction compared to other cells (Figure 2B). As cell density increased, E-cadherin expression increased and became enriched at sites of cell–cell contact in HuH-7, HepG2 and LM3 cells (Supplementary Figures S1A,B). However, in SK-HEP-1 cells, E-cadherin was located in the cytoplasm even when approaching high confluence (Supplementary Figure 1A). To better mimic an *in vivo* context, we cultured HCC cells into 3D multicellular tumor spheroids. Consistent with the results from 2D cell culture analysis, RSL3 triggered more prominent cell death (measured positive by SYTOX Green staining) in spheroids formed by HuH-7 cells and HepG2 cells (Figure 2E). Upon RSL3 treatment, cell viability was also most significantly reduced in HuH-7 and HepG2 spheroids (Figure 2F), whereas LM3 and SK-HEP-1 spheroids showed resistance.

### Oncogenic Activation of YAP Signaling Pathway Sensitizes Ferroptosis in HCC Cells

The pro-oncogenic transcriptional co-activator YAP is one of the downstream effectors suppressed by Merlin-Hippo signaling (Li, Cooper et al., 2012; Varelas and Wrana 2012). In addition, previous studies and our experiments have shown an exquisite correlation between YAP/TAZ activity and cell-density-dependent ferroptosis in different tumor cell lines (Wu, Minikes et al., 2019; Yang, Ding et al., 2019; Yang, Huang et al., 2020). These observations prompted us to perform a series of functional experiments to determine whether YAP promotes ferroptosis.

**FIGURE 3:**
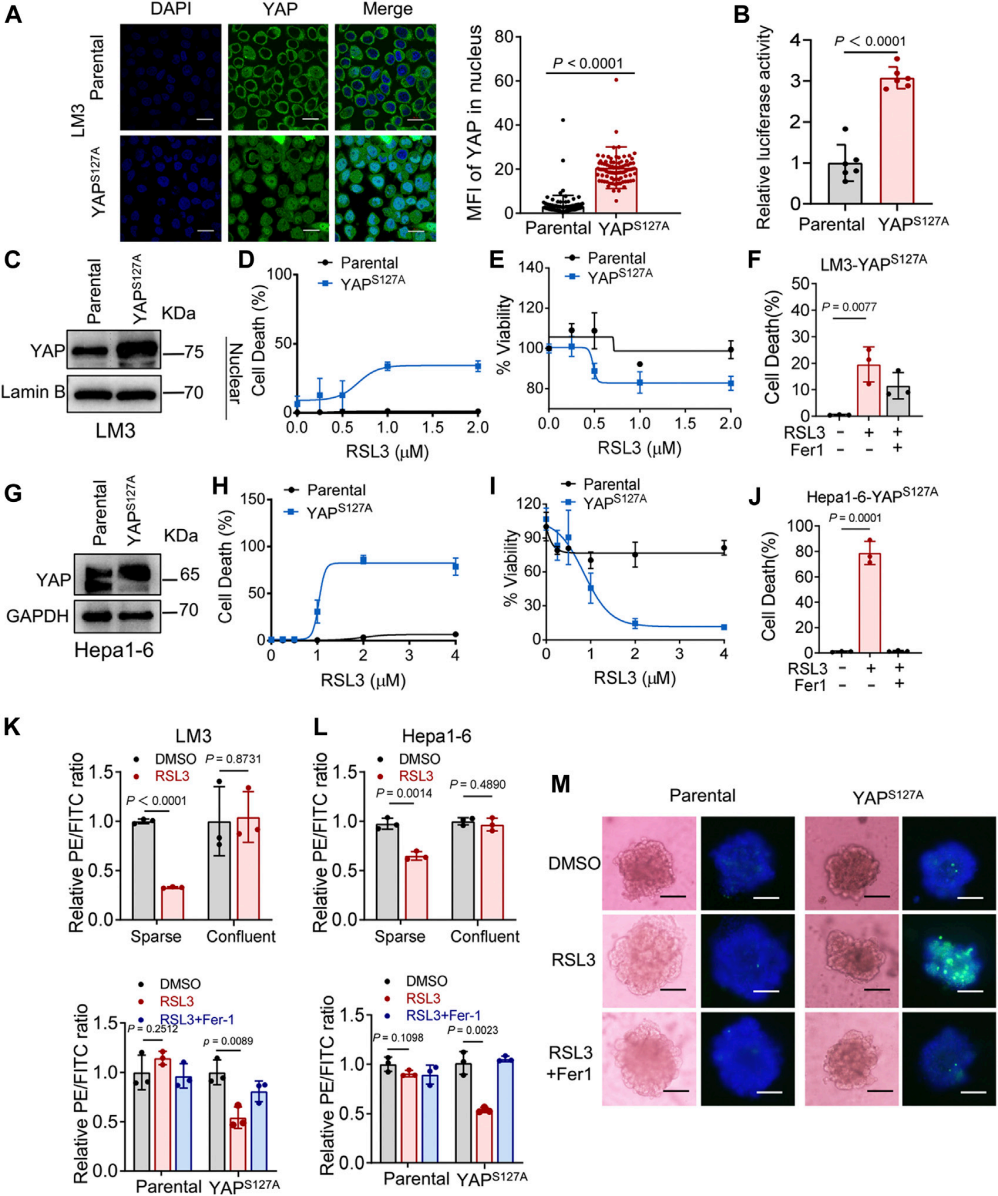
YAP activation sensitizes ferroptosis in HCC cells. **(A)** LM3 cells were infected with retroviruses encoding Flag-YAP^S127A^. Localization of YAP (green) was determined by immunofluorescence. Right, the expression of YAP in nucleus was quantified by ImageJ. Scale bars, 20 μm. Parental, *n* = 99 cells from 3 images, YAP^S127A^, *n* = 90 cells from 3 images. **(B)** YAP/TEAD transcriptional activity in confluent LM3 cells expressing YAP^S127A^ was measured by a luciferase assay using the 8xGTIIC-luciferase reporter. *n* = 6 biologically independent samples per condition. **(C)** Levels of YAP in the nucleus of LM3 cells were analyzed by western blot. **(D,E)** Confluent parental LM3 cells and YAP^S127A^-overexpressing cells were treated with RSL3. Cell death was measured following 12-h treatment of RSL3 and cell viability was assayed following 18 h treatment of RSL3 with indicated concentrations. *n* = 3 biologically independent samples per condition. **(F)** Confluent LM3-YAP^S127A^ cells were treated with or without RSL3 (1 μM) or Fer-1 (1 μM) as indicated for 15 h. Cell death was measured. n = 3 biologically independent samples per condition. **(G)** Levels of YAP in confluent parental Hepa1-6 cells and YAP^S127A^-overexpressing cells were analyzed by Western blot. **(H,I)** Cell death and cell viability in confluent parental Hepa1-6 cells and YAP^S127A^-overexpressing cells treated with RSL3 with indicated concentrations. **(H)** *n* = 3 biologically independent samples per condition. **(I)** *n* = 2 biologically independent samples per condition. **(J)** Cell death of confluent Hepa1-6-YAP^S127A^ cells treated with or without RSL3 (1 μM) or Fer-1 (1 μM) as indicated for 15 h *n* = 3 biologically independent samples per condition. **(K)** Up, lipid ROS production in LM3 cells, cultured with indicated cell density and treated with 2 μM RSL3 for 6 h. Bottom, lipid ROS production in parental and YAP^S127A^-overexpressing confluent LM3 cells treated with 2 μM RSL3 for 6 h, with or without Fer-1 (1 μM). **(L)** Up, lipid ROS production in Hepa1-6 cells, cultured with indicated cell density and treated with 2 μM RSL3 for 6 h (the decreasing PE/FITC ratio means increasing lipid peroxidation). Bottom, lipid ROS production in parental and YAP^S127A^-overexpressing confluent Hepa1-6 cells treated with 2 μM RSL3 for 6 h, with or without Fer-1 (1 μM). *n* = 3 biologically independent samples per condition. **(M)** Three-dimensional spheroids generated from parental and YAP^S127A^-overexpressing LM3 cells were treated with 2 μM RSL3 for 20 h. Dead cells were stained by SYTOX green, while all nuclei were stained with Hoechst 33342 (blue). Scale bars, 200 μm.

**FIGURE 4:**
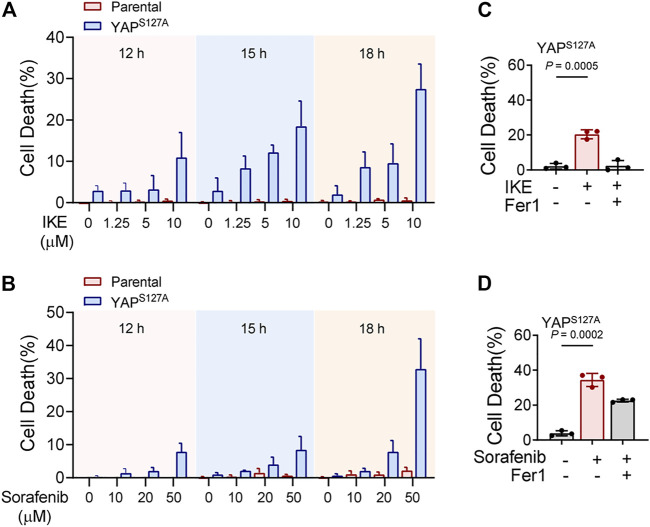
YAP activation sensitizes ferroptosis triggered by pharmacological inhibition of system xc-cystine/glutamate antiporter in HCC cells. **(A)** Parental LM3 cells and YAP^S127A^-overexpressing cells were treated with IKE with indicated concentrations. At different time points after IKE treatment, cell death was measured. **(B)** Cell death of parental LM3 cells and YAP^S127A^-overexpressing cells treated with sorafenib for indicated time. **(C)** Inhibition of 10 μM IKE-induced cell death by Fer-1 in YAP^S127A^-overexpressing LM3 cells. **(D)** Inhibition of 50 μM sorafenib-induced cell death by Fer-1 in YAP^S127A^-overexpressing LM3 cells. **n** = 3 biologically independent samples per condition.

**FIGURE 5:**
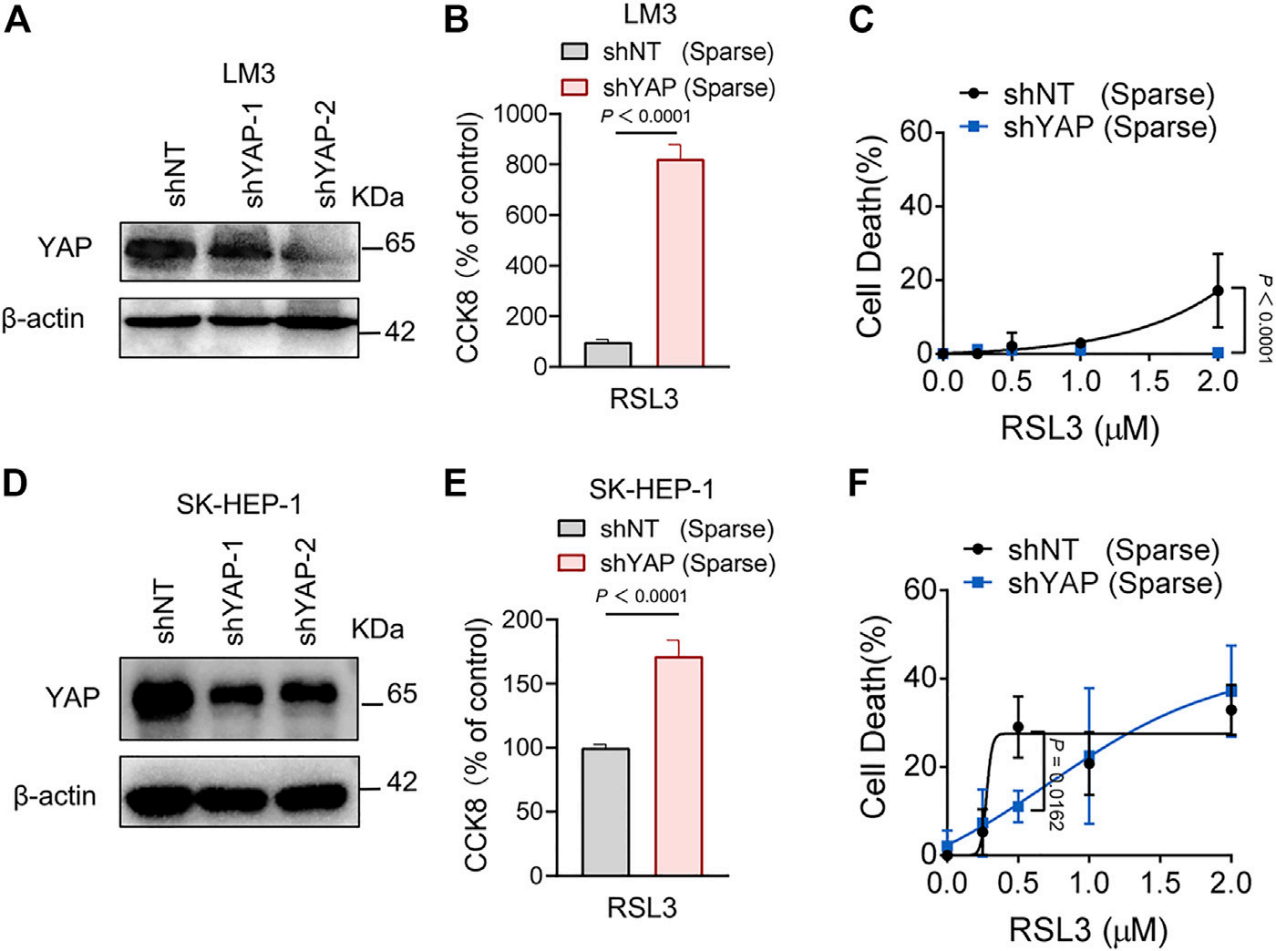
Knockdown of YAP inhibits ferroptosis in HCC cells. **(A)** LM3 were infected with shYAP-1 or shYAP-2 lentivirus and selected with puromycin. Knockdown efficiency was confirmed by western blot. **(B)** Sparse LM3 cells expressing shNT or shYAP as indicated were treated with 2 µM RSL3. Cell viability was determined using the Cell Counting Kit 8 (CCK8). *n* = 5 biologically independent samples per condition. **(C)** Sparse LM3 cells expressing shNT or shYAP were treated with RSL3 with indicated concentrations and cell death was measured. *n* = 3 biologically independent samples per condition. **(D)** Knockdown efficiency of YAP in SK-HEP-1 cells was confirmed by western blot. **(E)** Sparse SK-HEP-1 cells expressing shNT or shYAP were treated with 1 µM RSL3 and cell viability was determined with CCK8. *n* = 7 biologically independent samples per condition. **(F)** Sparse SK-HEP-1 cells expressing shNT or shYAP were treated with RSL3 and cell death was measured. *n* = 3 biologically independent samples per condition.

First, we utilized the constitutively active YAP mutant, S127A (serine-127 mutated to alanine). This mutant cannot be phosphorylated by Lats1/2 at the S127 residue and thus is retained in the nucleus to exert its transcriptional co-regulatory activity. Compared with the parental control, LM3 cells ectopically expressing the YAP^S127A^ mutant were unable to exclude the YAP mutant from the nucleus, even when cultured at a high density (Figure 3A). YAP is functionally activated with overexpression of YAP^S127A^, measured by an 8xGTIIC-luciferase reporter assay that monitors the transcriptional activity of YAP with its primary binding partners, the TEAD family of transcription factors (Figure 3B). LM3 or Hepa1-6 HCC cells that express YAP^S127A^ were markedly more sensitive to ferroptosis induced by RSL3 (Figures 3C–J). YAP^S127A^ also enhanced lipid ROS generation (Figures 3K,L). Consistently, the YAP ^S127A^ mutant also sensitized ferroptosis in 3D tumor spheroids derived from LM3 cells (Figure 3M). We found that YAP ^S127A^ mutant enhanced ferroptosis induced by imidazole ketone erastin (IKE) (Figure 4A), a potent and metabolically stable analog of erastin that has been validated for *in vivo* use, as well as sorafenib (Figure 4B). Treatment with the lipid peroxide-trapping agent ferrostatin-1 (Fer-1) prevented cell death triggered by IKE in LM3 cells with overexpression of YAP ^S127A^ (Figures 4C,D). Notably, Fer-1 didn’t completely prevent cell death triggered by sorafenib, suggesting the existence of ferroptosis-independent cell death. Furthermore, knockdown of YAP in sparse LM3 and SK-HEP-1 cells conferred resistance to ferroptosis (Figure 5). These results confirm the role of oncogenic activation of YAP signaling in sensitizing ferroptosis in HCC cells.

### Identification of ALOXE3 as an Essential Factor of YAP-Sensitized Ferroptosis

**FIGURE 6:**
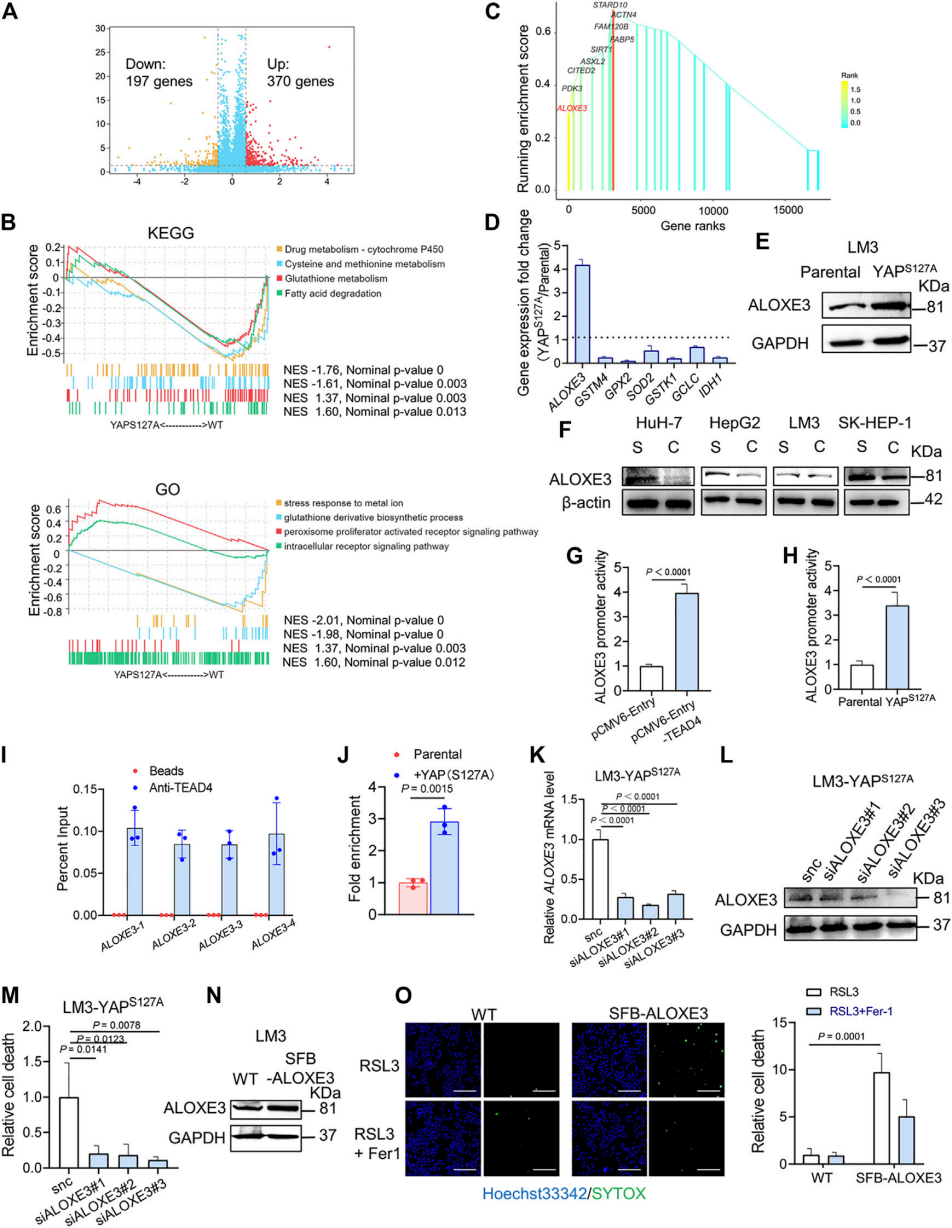
ALOXE3 in regulating YAP-mediated ferroptosis in human HCC cell lines. **(A)** YAP^S127A^ mutant and parental LM3 cells were analyzed by RNA sequencing. Identification of differentially expressed genes is illustrated in a volcano plot. **(B)** GSEA enrichment plot showing the enrichment of genes associated with pathways involved in ferroptosis. **(C)** Genes comprising the leading edge of Enrichment score in the category of peroxisome proliferator activated receptor signaling pathway in YAP ^S127A^-overexpressing cells. **(D)** Relative expression of the genes associated with ferroptosis regulation. *n* = 3 biologically independent samples per condition. **(E)** The expression of ALOXE3 in YAP^S127A^-overexpressing and parental LM3 cells was tested by western blot. **(F)** The expression of ALOXE3 in sparse and confluent HCC cells. S, sparse. C, confluent. **(G)** A dual-luciferase reporter assay was performed in LM3 cells, which were co-transfected with ALOXE3 promoter (pGL-ALOXE3) and TEAD plasmid (pCMV-Entry-TEAD4) or empty plasmid (pCMV-Entry). **(H)** A dual-luciferase reporter assay was performed in YAP^S127A^-overexpressing and parental LM3 cells, which were transfected with ALOXE3 promoter (pGL-ALOXE3). *n* = 3 biologically independent samples per condition. **(I)** ChIP analysis of TEAD4 binding to the ALOXE3 promoter in LM3 cells using beads control or an anti-TEAD4 antibody. Values are percentage of input. *n* = 3 biologically independent samples per condition. **(J)** ChIP analysis monitoring the occupancy of TEAD4 on the ALOXE3 promoters in parental or YAP^S127A^-overexpressing LM3 cells. Enrichment was calculated based on qPCR relative to the beads control. *n* = 3 biologically independent samples per condition. **(K,L)** LM3-YAP^S127A^ cells were transfected with siALOXE3. Knockdown efficiency was confirmed by qPCR **(K)** and western blot **(L)**. *n* = 3 biologically independent samples per condition. **(M)** Confluent LM3-YAP^S127A^ cells transfected with siALOXE3 were treated with RSL3 and cell death was measured. **(N)** LM3 cells were transfected with a plasmid encoding ALOXE3 (SFB-ALOXE3). Levels of ALOXE3 were analyzed by western blot. **(O)** Confluent LM3 cells transfected with SFB-ALOXE3 were treated with RSL3 and cell death was measured with SYTOX. Scale bars, 200 μm *n* = 3 biologically independent samples per condition.

To investigate the mechanism of YAP activation in sensitizing ferroptosis, we performed RNA-sequencing in parental and YAP ^S127A^ mutant-overexpressing LM3 cells. In total, 567 differentially expressed genes were identified, including 370 upregulated and 197 downregulated genes in YAP ^S127A^ mutant-overexpressing LM3 cells (Figure 6A). In order to functionally annotate the expression differences between the 2 groups, we performed a gene set enrichment analysis (GSEA) and selected the signaling pathways related to ferroptosis for further analysis (Figure 6B). YAP ^S127A^ mutant-overexpressing LM3 cells showed a significant enrichment of genes in categories related to peroxisome proliferator activated receptor signaling pathway, while parental cells were strongly characterized by the expression of genes involved in cysteine and methionine metabolism and glutathione metabolism. Among genes comprising the leading edge of Enrichment score in the category of peroxisome proliferator activated receptor signaling pathway in YAP ^S127A^-overexpressing cells compared with parental cells, ALOXE3 belongs to the mammalian lipoxygenase family (Figure 6C), which has been revealed as a ferroptosis regulator (Yang, Kim et al., 2016; Xia, Liu et al., 2020; Yang, Liu et al., 2021). Based on publicly available ENCODE TEAD4 ChIP-seq datasets (GSM1614035, GSM1010860, GSM1010868, GSM1010772 and GSM1010875), we found that ALOXE3 is a putative YAP-TEAD4 gene target in various cancer cell lines. As shown in Figures 6D,E, the expression of ALOXE3 increased upon YAP ^S127A^ overexpression, suggesting that ALOXE3 might be transcriptionally regulated. As cell density increased, the level of ALOXE3 decreased in HuH-7, HepG2 and SK-HEP-1 cells (Figure 6F). To validate the contribution of YAP activation on ALOXE3 expression, the transcriptional regulation of ALOXE3 was investigated using a dual-luciferase reporter assay system. pGL3-ALOXE3 (−3000/+250) was co-transfected with pCMV6-Entry-TEAD4 into LM3 cells. As shown in Figure 6G, transfection of TEAD4 plasmid significantly increase the luciferase activity compared to cells transfected with empty plasmid. In addition, co-transfection of YAP ^S127A^ and the ALOXE3 promoter region also led to an increase in luciferase activity compared to parental cells (Figure 6H). ChIP analysis of TEAD4 binding to the ALOXE3 promoter in LM3 cells using beads control or an anti-TEAD4 antibody. To validate the TEAD4 binding to the regulation regions of ALOXE3 in HCC cells, we did ChIP assay with anti-TEAD4 antibody. We found that TEAD4 binds to the promoter regions of ALOXE3 genes in parental LM3 cells (Figure 6I), and the binding was enhanced by overexpression of YAP^S127A^ (Figure 6J). Effective knockdown of ALOXE3 protected YAP ^S127A^-overexpressing cells from ferroptosis upon RSL3 treatment (Figures 6K−M), while overexpression of ALOXE3 restored ferroptosis induced by RSL3 in confluent cells (Figures 6N,O). These data indicate that ALOXE3 is essential for YAP-sensitized ferroptosis.

### Correlation of YAP With ALOXE3 in Human HCC Tissues

**FIGURE 7:**
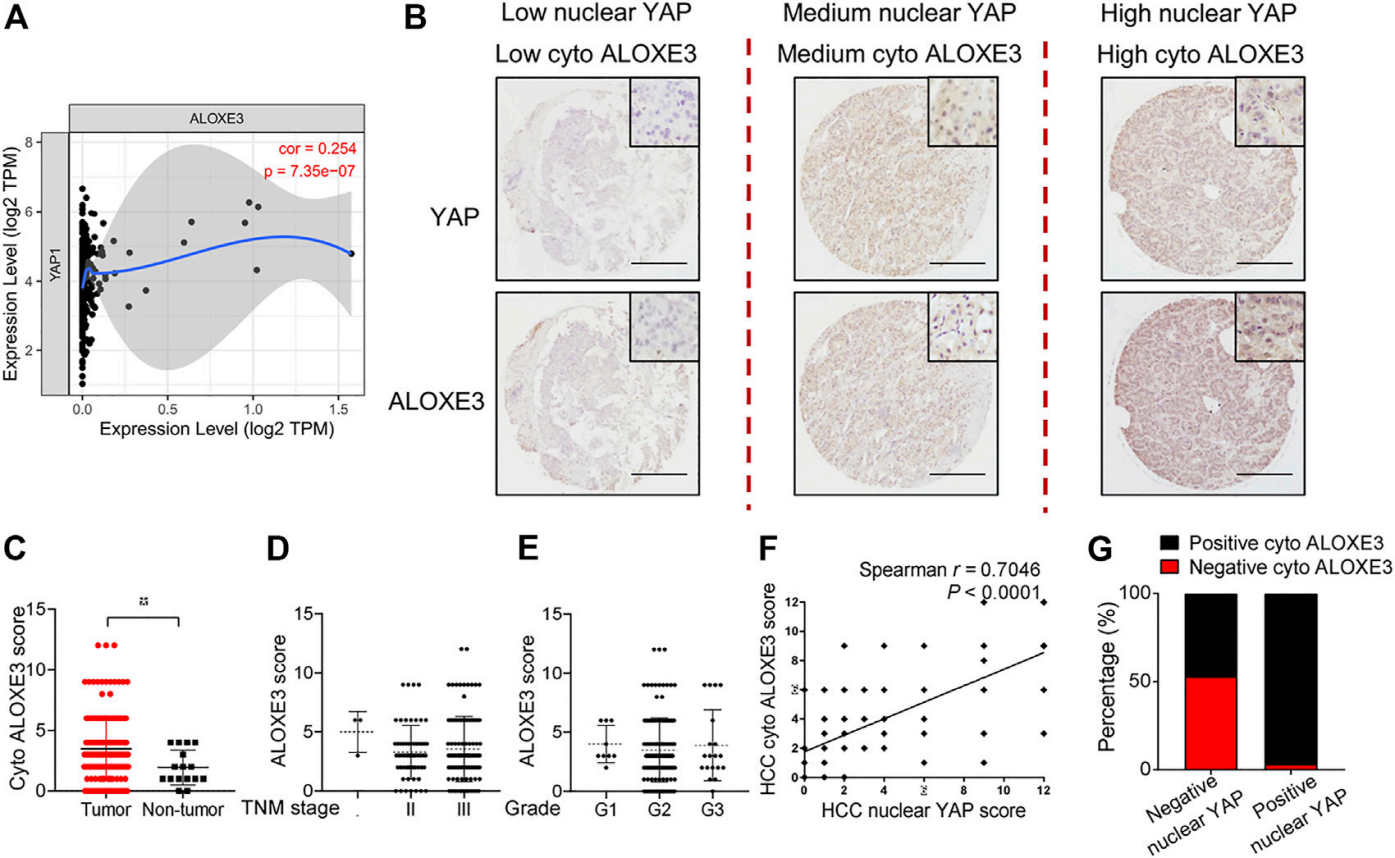
The correlation of YAP with ALOXE3 in HCC. **(A)** The correlation between expression levels of YAP and ALOXE3 in HCC tissues were analyzed using the TIMER database. **(B)** Representative images of immunohistochemical staining of YAP and ALOXE3 in HCC tissues and normal liver tissue on tissue microarray sections are shown. Scale bars, 200 μm. **(C)** Expression scores of ALOXE3 in HCC tissues (*n* = 174) and normal liver tissue (*n* = 16) assessed by immunohistochemistry. **(D,E)** ALOXE3 expression in tumor tissues of HCC patients with different tumor grades or with different TNM classification. I, *n* = 3 patients. II, *n* = 61 patients. III, *n* = 110 patients. G1, *n* = 9 patients. G2, *n* = 140 patients. G3, *n* = 20 patients. **(F)** Positive correlation between the nuclear YAP score and the ALOXE3 score in human HCC tissues (*n* = 174). **(G)** Percentage of tumors with positive or negative staining of nuclear YAP or ALOXE3.

We first analyzed the correlation between YAP mRNA expression and ALOXE3 in hepatocellular carcinoma using data from the TCGA. The mRNA expression of YAP is positively correlated with ALOXE3 (Figure 7A). We then performed immunohistochemistry to investigate the correlation between nuclear YAP and ALOXE3 in human HCC tissues and normal liver tissue using a high-throughput tissue microarray analysis (Figure 7B). As shown in Figure 7C, ALOXE3 level was significantly higher in HCC patients than in normal liver tissues (*p* < 0.001) but showed no significant correlation with TNM stages and grades of HCC (Figures 7D,E). Statistical analysis showed that the level of nuclear YAP in the primary HCC lesions was positively correlated with the expression of ALOXE3 **(** Figure 7F) (*r =* 0.7046, *p <* 0.0001). Among nuclear YAP negative tumors samples, 53.13% tumor samples exhibited negative staining of ALOXE3. However, among nuclear YAP positive tumors, only 3.21% exhibited negative staining of ALOXE3 (Figure 7G), suggesting the potential regulation of ALOXE3 by YAP.

### Combination of YAP Activation With Ferroptosis Induction With Sorafenib Leads to Tumor Regression *in Vivo*

**FIGURE 8:**
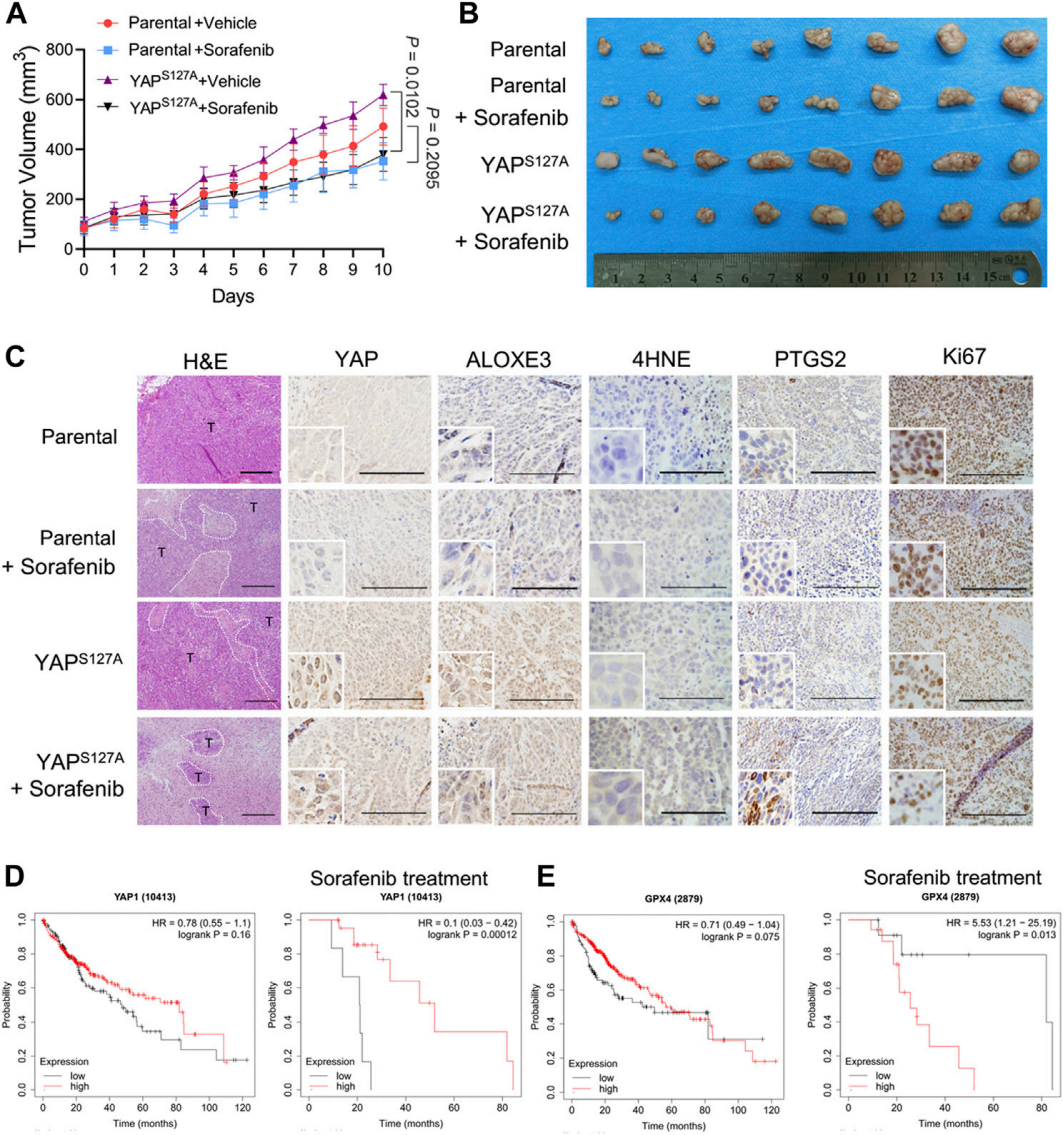
The cancer therapeutic potential of ferroptosis induction with sorafenib in HCC with enhanced activation of YAP. Parental or YAP^S127A^-overexpressing LM3 cells (2 × 10^6^) were injected subcutaneously into nude mice. The average volume of tumors was allowed to reach ∼100 mm^3^, mice bearing tumors were orally administered with 50 mg/kg sorafenib daily for 10 days. **(A)** Growth curves of tumors of each group. *n* = 8, on the linear scale for actual tumor size. **(B)** Images of resected tumors from mice xenografted with parental or YAP^S127A^-overexpressing LM3 cells. **(C)** Representative H&E and immunostaining images of YAP, ALOXE3, 4HNE, PTGS2 and Ki67. Scale bars for 4HNE, 100 μm. Scale bars for others, 200 μm. **(D)** Comparison of the survival curves of YAP with high and low expression in HCC with or without sorafenib treatment in the GEPIA database. **(E)** Comparison of the survival curves of GPX4 with high and low expression in HCC with or without sorafenib treatment in the GEPIA database.

To explore the cancer therapeutic potential of ferroptosis induction in HCC with enhanced activation of YAP, we used parental LM3 cells and cells ectopically expressing the YAP^S127A^ mutant to produce subcutaneous xenograft tumors in athymic nude mice. In mice xenografted with these cells, we allowed the average volume of tumors to reach ∼100 mm^3^, and then started ferroptosis induction by sorafenib. We found that overexpression of YAP^S127A^ mutant rendered xenograft tumors significantly more sensitive to sorafenib (Figures 8A,B). H&E staining showed that sorafenib administration resulted in significantly larger zones of coagulation necrosis in tumors generated by YAP^S127A^ mutant LM3 cells compared with tumors generated by parental cells (Figure 8C). As confirmed by immunohistochemical analysis, levels of YAP in nucleus and ALOXE3 in cytoplasm were greatly increased in tumor tissues generated by YAP^S127A^ mutant LM3 cells. Immunohistochemical analysis of 4HNE and PTGS2, markers of lipid peroxidation and ferroptosis, supported the synergistic effect of sorafenib in inducing tumor ferroptosis in HCC cells with enhanced activation of YAP *in vivo* (Figure 8C). Ki67 expression, indicative of cell proliferation, was reduced (Figure 8C).

To further investigate the correlation of YAP expression and the survival rate of patients with HCC, we used the survival information retrieved by the GEPIA database to further validate our results analysis. We found that YAP level was not significantly associated with the overall survival of patients (Figure 8D). However, for patients who had been treated with sorafenib, patients with tumors exhibiting high level of YAP had better survival rates than those with tumors exhibiting low YAP expression level (Figure 8D). A significantly negative association between GPX4 status and the overall survival rate was also observed in HCC patients with sorafenib treatment (Figure 8E). Thus, enhancing the response of HCC cells to sorafenib-induced ferroptosis may become an effective strategy to improve the antitumor performance of sorafenib *in vivo.*

## Discussion

Ferroptosis can be initiated by cystine/cysteine deprivation or the loss of glutathione peroxidase-4 (GPX4) activity (Yang, SriRamaratnam et al., 2014; Gao, Monian et al., 2015). Erastin, an inhibitor of the cystine/glutamate antiporter system Xc-, blocks cystine import and downstream glutathione synthesis, leading to dysregulated cellular redox homeostasis, accumulated lipid peroxides/ROS, and the induction of cell death (Dixon, Lemberg et al., 2012). GPX4 is a glutathione-dependent enzyme in mammalian cells that catalyzes the clearance of lipid ROS. Pharmacological inhibition of GPX4 enzymatic activity can also trigger ferroptosis (Yang, SriRamaratnam et al., 2014). Sorafenib is an orally administered multi-kinase inhibitor capable of facilitating apoptosis, mitigating angiogenesis and suppressing tumor cell proliferation (Llovet, Ricci et al., 2008). Although sorafenib is currently being used as an effective first-line therapy for late-stage HCC, the development of drug resistance to sorafenib is becoming increasingly common (Tang, Chen et al., 2020). Sorafenib also induces ferroptosis by inhibition of the xc-amino acid antiporter (Dixon, Patel et al., 2014), and the inhibition of ferroptosis is involved in sorafenib resistance in HCC (Sun, Niu et al., 2016). Notably, a recent study suggests that sorafenib does not induce ferroptosis in a series of tumor cell lines unlike the cognate system xc− inhibitors sulfasalazine and erastin (Zheng, Sato et al., 2021). Consistently, we found that sorafenib failed to induce ferroptosis in parental LM3 cells. We also found that ferroptosis inducer could only partially inhibit cell death induced by sorafenib in YAP-activating LM3 cells, suggesting that YAP might enhance both ferroptotic cell death and ferroptosis-independent cell death. In conclusion, although enhancing the response of HCC cells to sorafenib-induced ferroptosis may become an effective strategy, whether sorafenib indeed qualifies as a robust *bona fide* system xc− inhibitor is proposed to be carefully assessed in the future.

As a transcriptional coactivator, YAP regulates the expression of multiple target genes through its interaction with the TEAD family of transcriptional factors (Zhao, Lei et al., 2008). YAP promote ferroptosis through upregulation of multiple ferroptosis modulators, including ACSL4, TFRC and SKP2 (Wu, Minikes et al., 2019; Yang, Lin et al., 2021). This study reveals the lipoxygenase ALOXE3 as another YAP-TEAD target gene that contributed to YAP-promoted ferroptosis. At the core of the ferroptosis process, PUFAs and PUFA-containing lipids are highly sensitive to non-enzymatic reactions (e.g., iron-dependent Fenton reaction) and oxidation by enzymes (e.g., ALOX and POR) (Tang, Chen et al., 2021). ALOX modulate the status of cellular PUFAs and thereby cells’ sensitivity to ferroptosis by contributing to the cellular accumulation of lipid hydroperoxides (Tang, Chen et al., 2021). The regulation of ferroptosis by ALOXE3 was revealed in several articles. ALOXE3 deficiency rendered GBM cells resistant to ferroptosis and promoting GBM cell survival, and knockdown of ALOXE3 in GBM cells fostered the orthotopic tumor growth in mice (Yang, Liu et al., 2021). Erastin-induced cell death was rescued by silencing either ALOX15B or ALOXE3 in HT1080 cells, which supported the hypothesis that lipoxygenases are required for ferroptosis under GSH-depleted condition (Yang, Kim et al., 2016). In addition, a novel ferroptosis inducer, talaroconvolutin A, enhanced ferroptosis in colorectal cancer cells by up-regulation of ALOXE3, which increases lipid peroxidation, the critical trigger for ferroptosis (Xia, Liu et al., 2020).

In various cancer types, YAP was found to induce cancer stem-like properties and promote tumor cell proliferation. Cancers can be parsed into binary YAP^on^ and YAP^off^ classes, and YAP states drive distinct adhesive, metabolic, genetic, and pharmaceutical vulnerabilities (Pearson, Huang et al., 2021). We found that sorafenib antitumor efficacy was improved in YAP-activated HCC cells with enhanced lipid ROS generation, since YAP activation sensitized confluent HCC cells to ferroptosis induced by sorafenib. Thus, the activation state of YAP may serve as a predictor of outcome for sorafenib treatment in patients with advanced HCC. As the Hippo–YAP signaling axis is frequently mutated in cancer, this study has clear implications for the application of sorafenib as a ferroptosis-inducer in other cancer types besides HCC. Worth mentioning, IKE, which also targets xc-amino acid antiporter to promote cystine/cysteine deprivation and induce ferroptosis, has been widely tested as an *in vivo* ferroptosis-inducing cancer therapeutic agent (Wu, Minikes et al., 2019; Zhang, Tan et al., 2019; Ye, Chaudhary et al., 2020) because of its strong potency, metabolic stability and safety *in vivo*. In our study, YAP-activated HCC cells showed increased vulnerability to IKE. Ferroptosis-inducers including IKE and sorafenib might have considerable benefits in overcoming cancer resistance to current treatments.

## Data Availability

The datasets presented in this study can be found in online repositories. The names of the repository/repositories and accession number(s) can be found below: NCBI BioProject PRJNA762931, https://www.ncbi.nlm.nih.gov/bioproject/PRJNA762931.

## Supplementary Material

Supplementary Figure S1 Localization and expression of E-cadherin in HuH-7, HepG2, LM3 and SK-HEP-1 cells. **(A)** HCC cells were cultured under sparse or confluent conditions. E-cadherin localization was assessed by immunofluorescence. Sparse, 3 × 10^4^/35 mm confocal dish. Confluent, 3 × 10^5^/35 mm confocal dish. Scale bars, 50 μm. **(B)** Expression of E-cadherin of HCC cells under sparse or confluent conditions detected by western blot.
