# Supplementary material for: Oncogenic Activation of YAP Signaling Sensitizes Ferroptosis of Hepatocellular Carcinoma via ALOXE3-Mediated Lipid Peroxidation Accumulation
Source: Front Cell Dev Biol. 2021 Dec 16;9:751593. doi: 10.3389/fcell.2021.751593 (PMC8717939; doi:10.3389/fcell.2021.751593)

Figure 1B

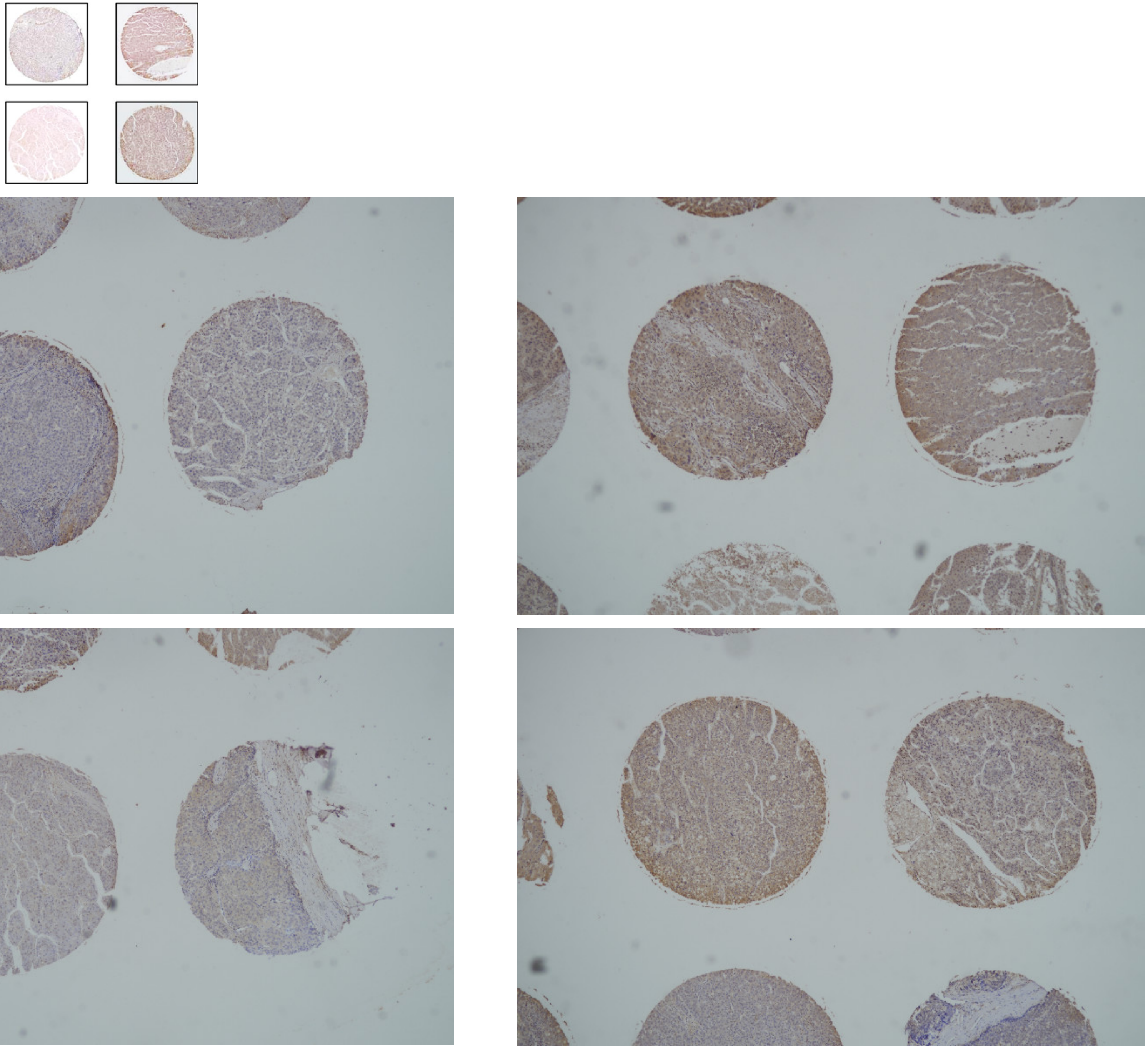

Figure 2A

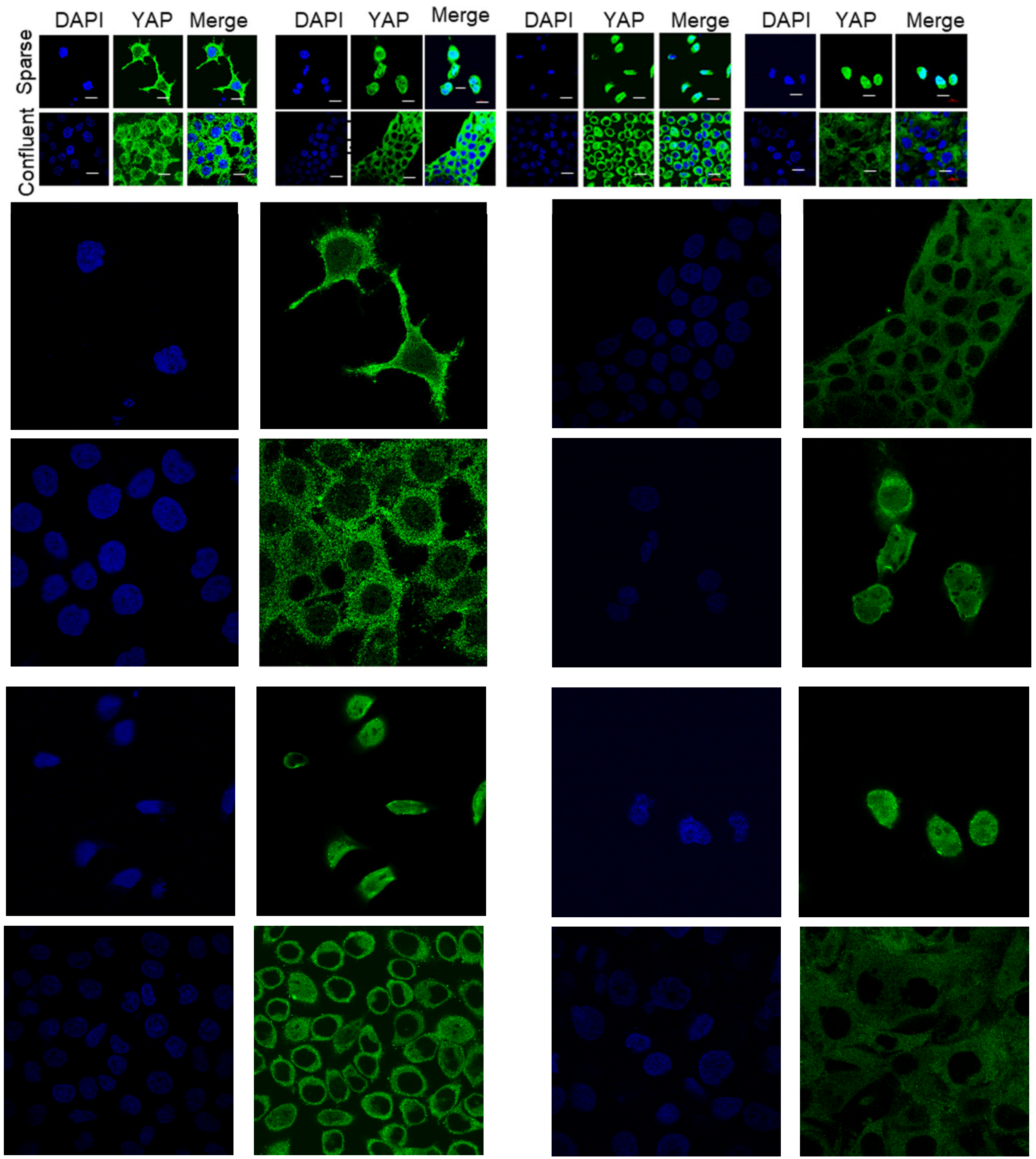
DAPI
YAP
Merge
DAPI
YAP
Merge
DAPI
YAP
Merge
DAPI
YAP
Merge
Sparse
Confluent

Figure 2E

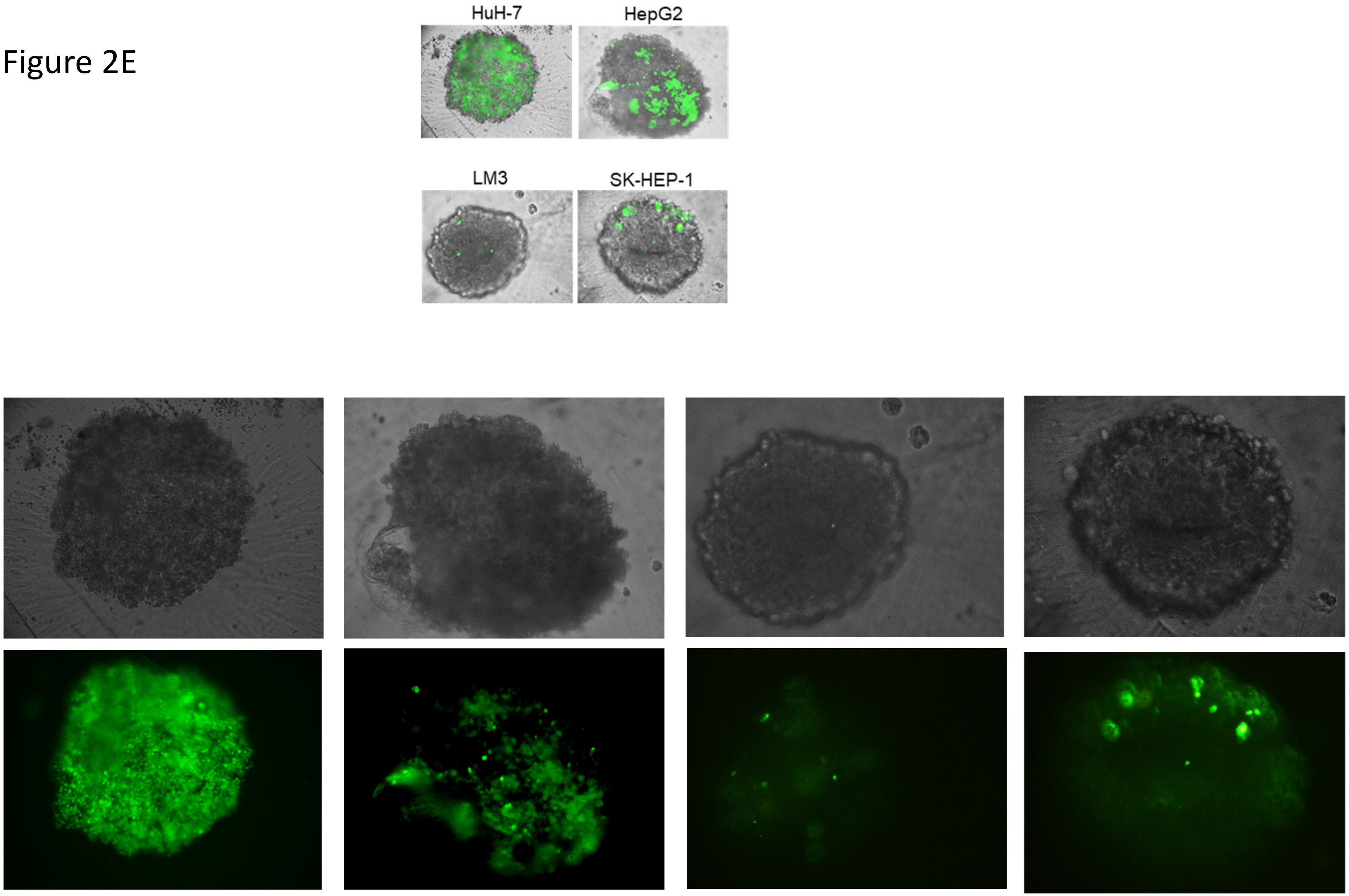

Figure 3A

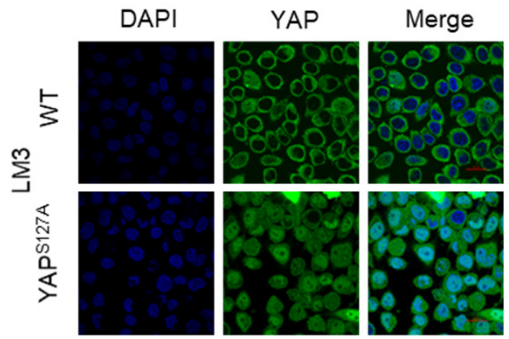

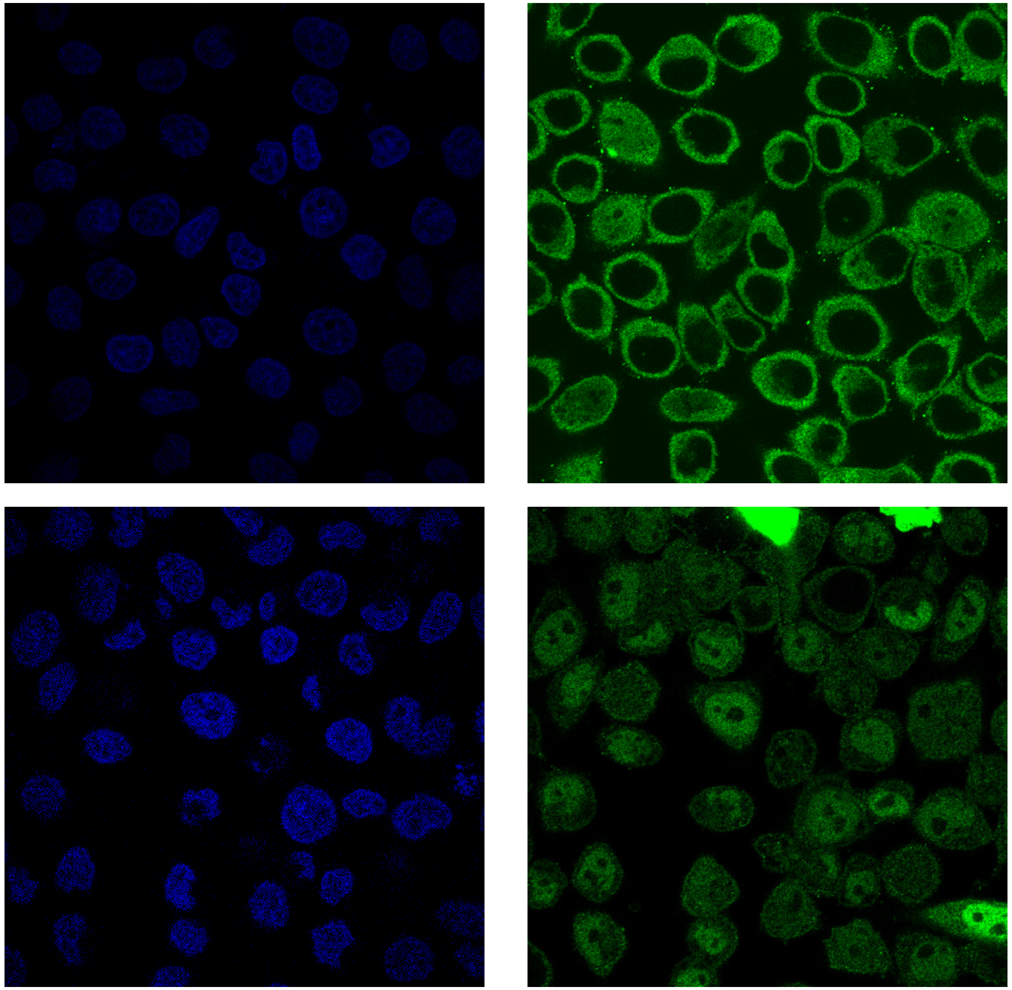

Figure 3C

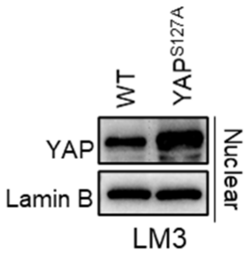
WT
YAP S127A
YAP
Lamin B
Nuclear
LM3

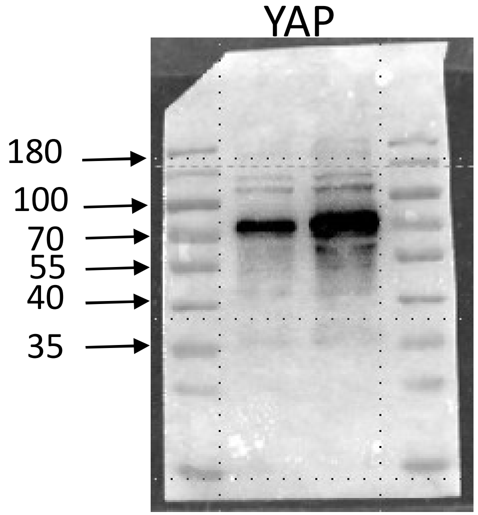
YAP
180
100
70
55
40
35

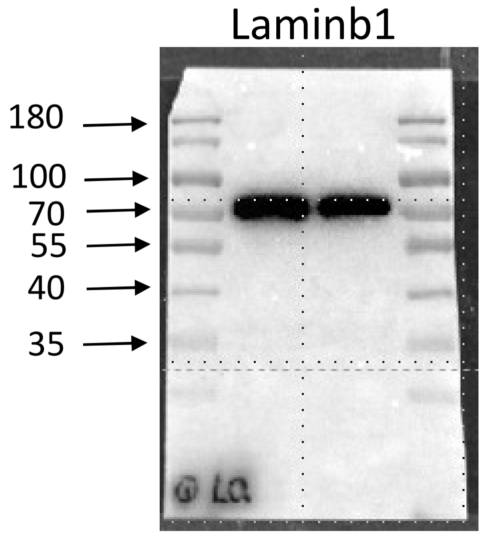
Laminb1
180
100
70
55
40
35

Figure 3G

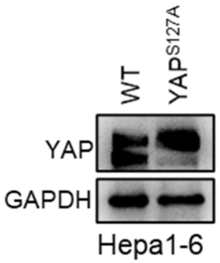

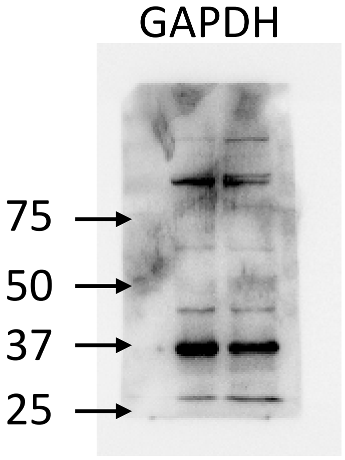

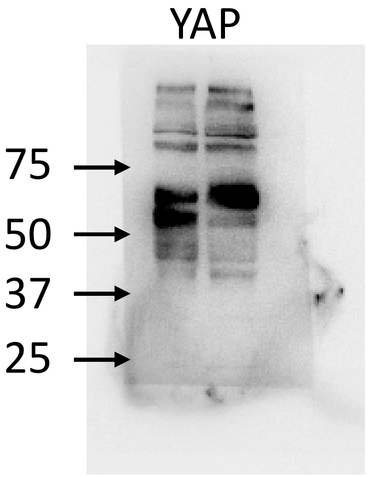

Figure 3M

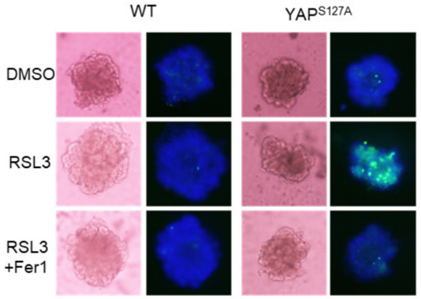

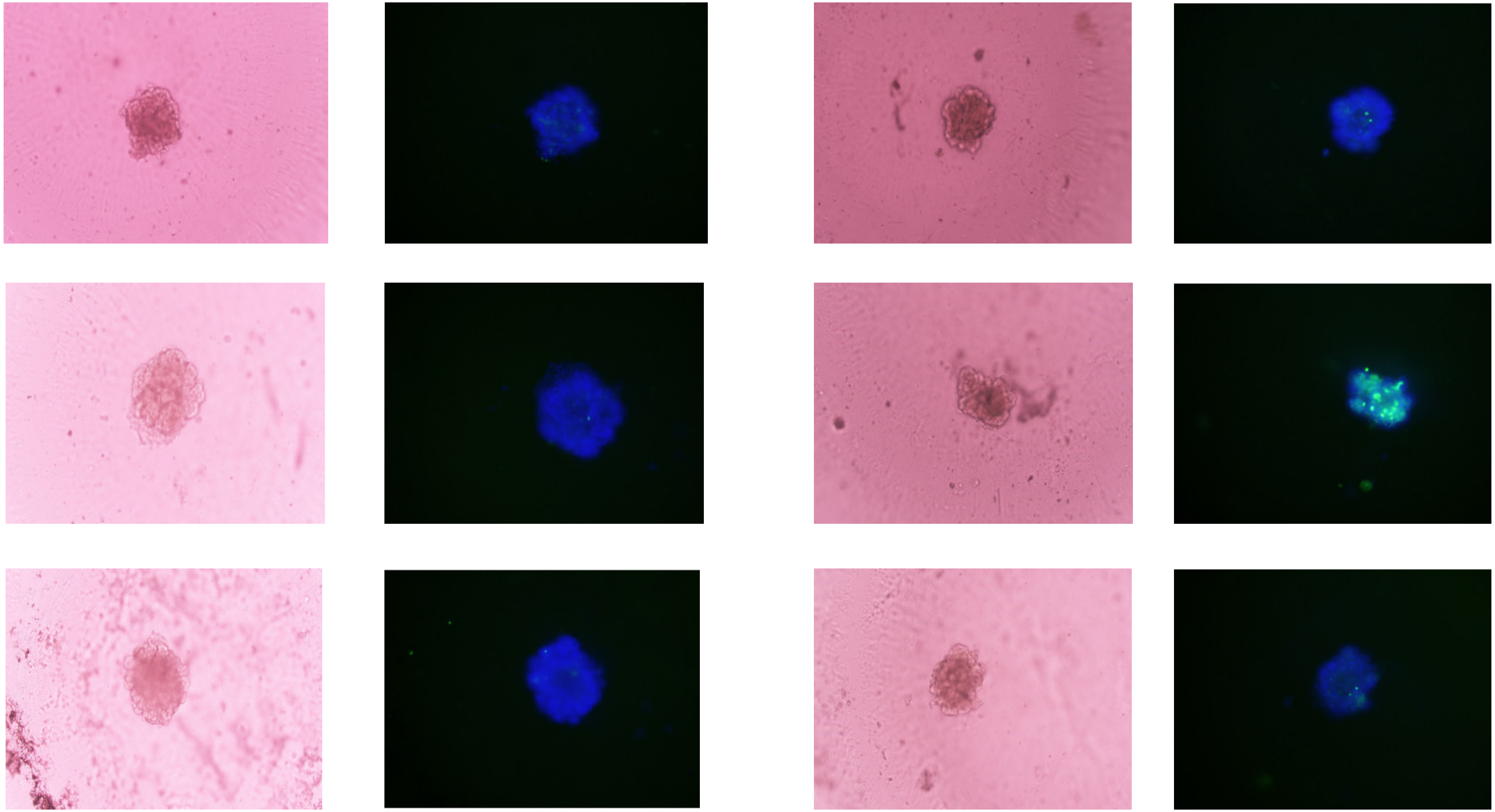

Figure 5A

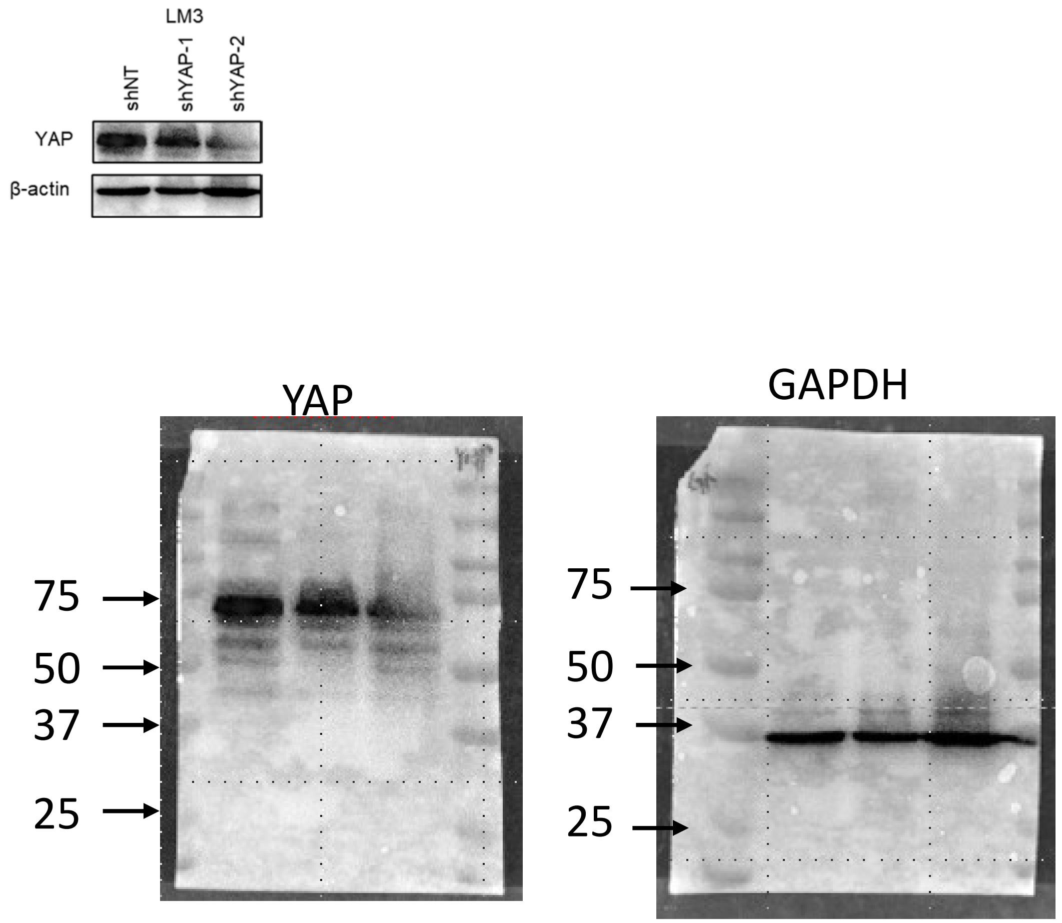
LM3
shNT
shYAP-1
shYAP-2
YAP
β-actin
YAP
75
50
37
25
GAPDH
75
50
37
25

Figure 5D

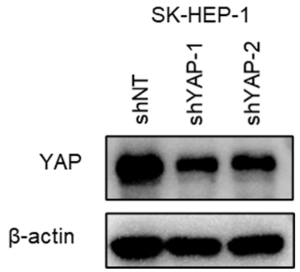
SK-HEP-1
shNT
shYAP-1
shYAP-2
YAP
β-actin

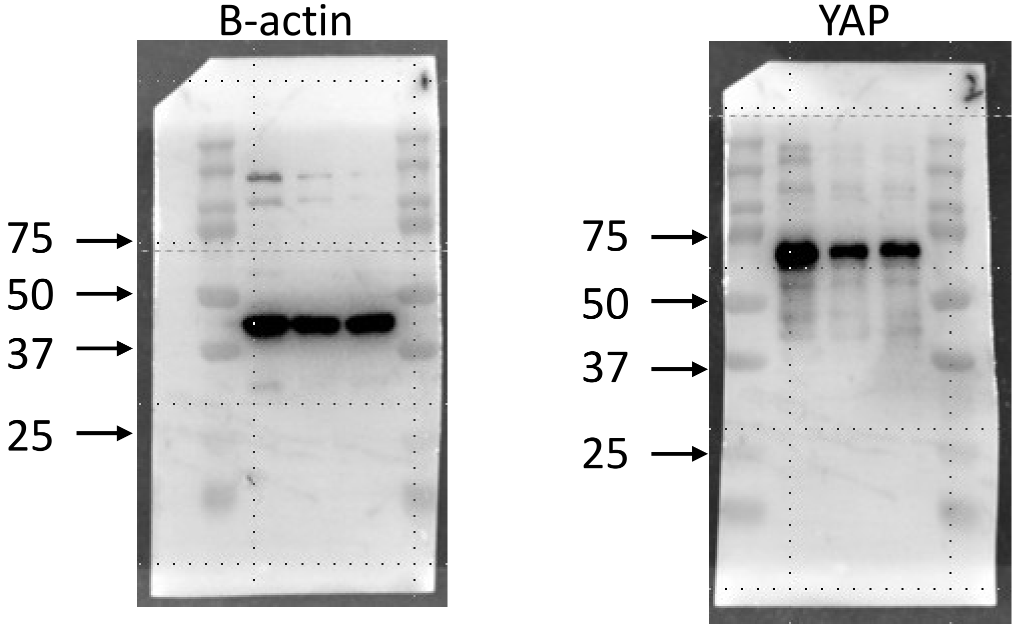
B-actin
75
50
37
25
YAP
75
50
37
25

Figure 6E

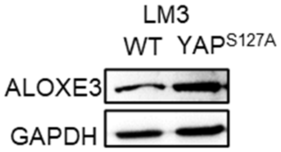

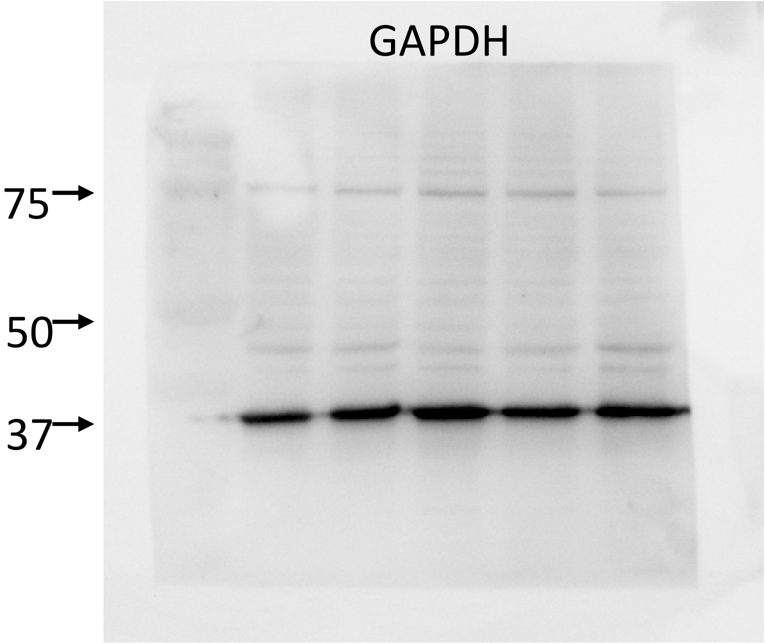

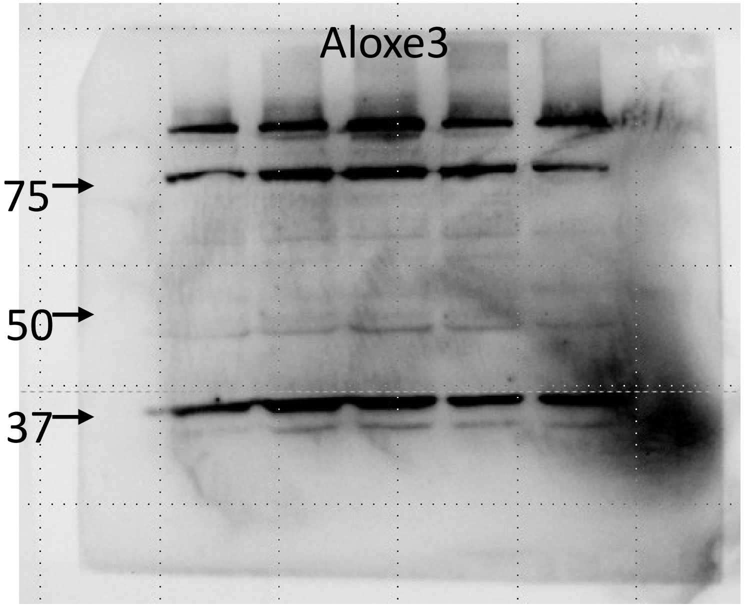

Figure 6F

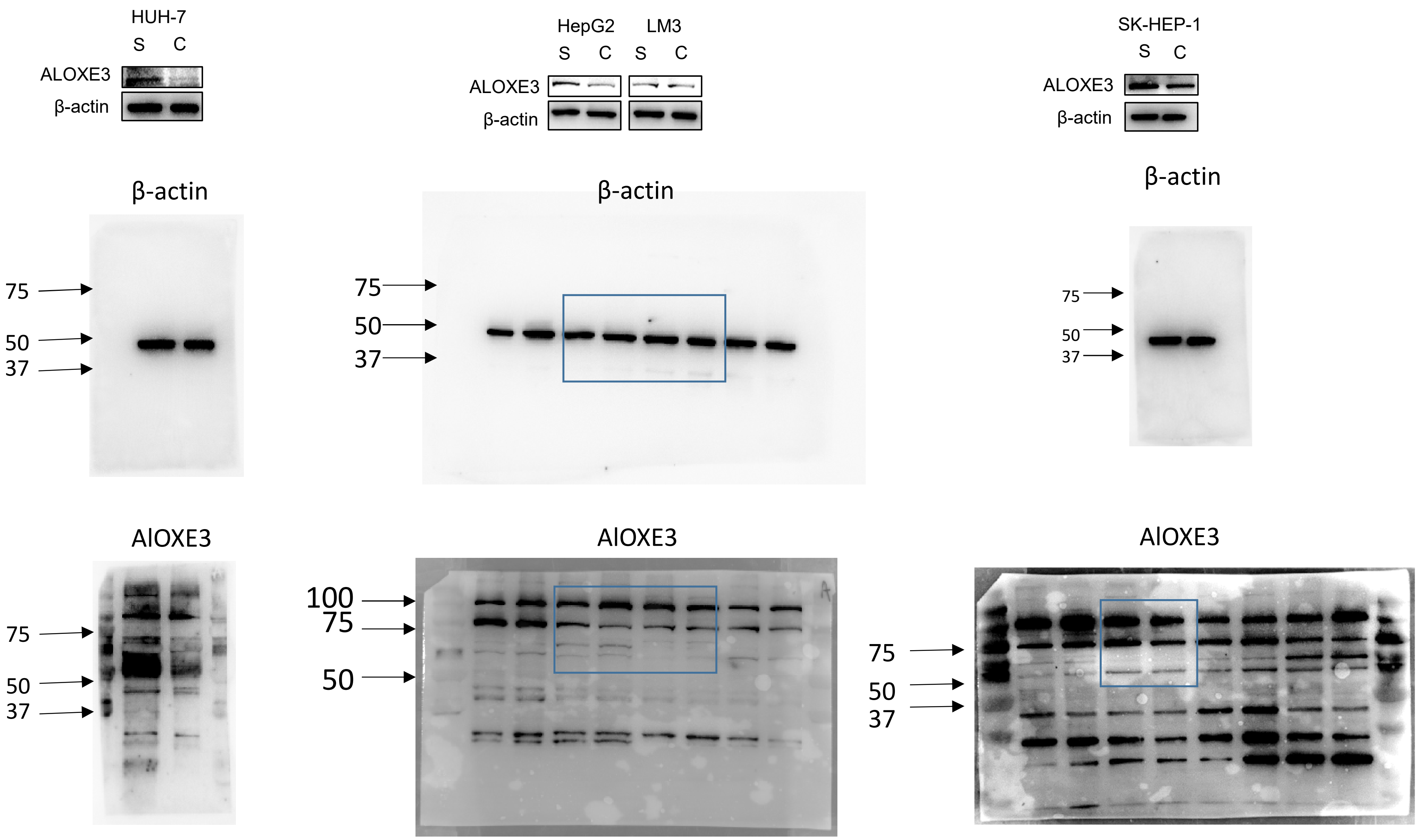

HUH-7
S C
ALOXE3
β-actin
HepG2 LM3
S C S C
ALOXE3
β-actin
SK-HEP-1
S C
ALOXE3
β-actin
β-actin
75
50
37
β-actin
75
50
37
β-actin
75
50
37
AlOXE3
75
50
37
AlOXE3
100
75
50
AlOXE3
75
50
37

Figure 6L

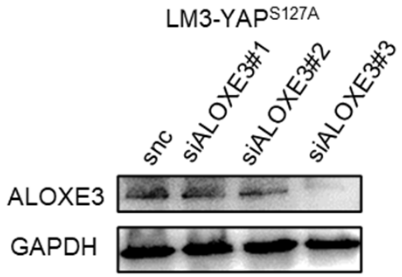
LM3-YAP^{S127A}
snc
siALOXE3#1
siALOXE3#2
siALOXE3#3
ALOXE3
GAPDH

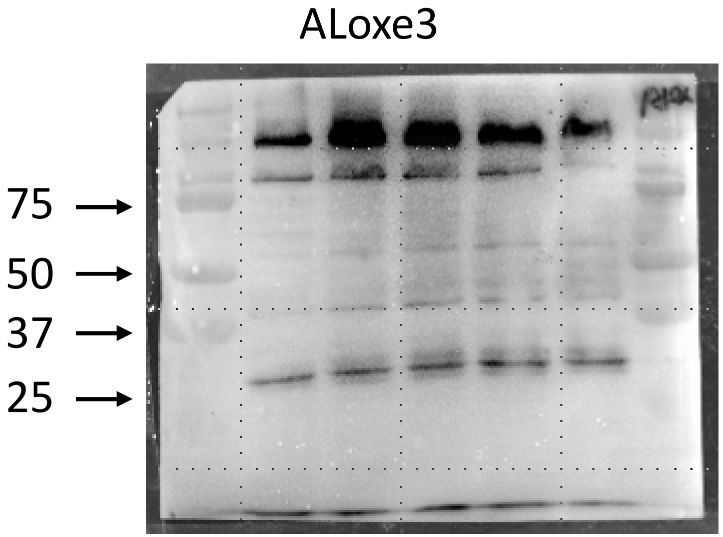
ALoxe3
75
50
37
25

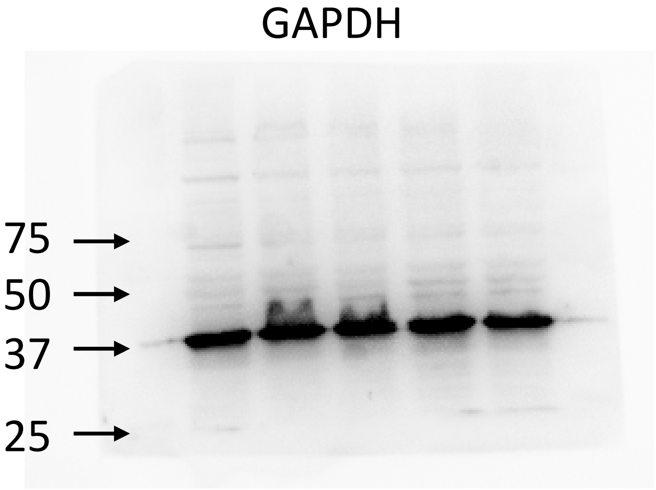
GAPDH
75
50
37
25

Figure 6N

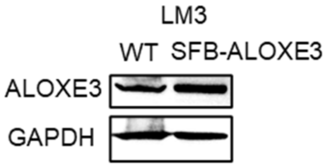
LM3
WT
SFB-ALOXE3
ALOXE3
GAPDH

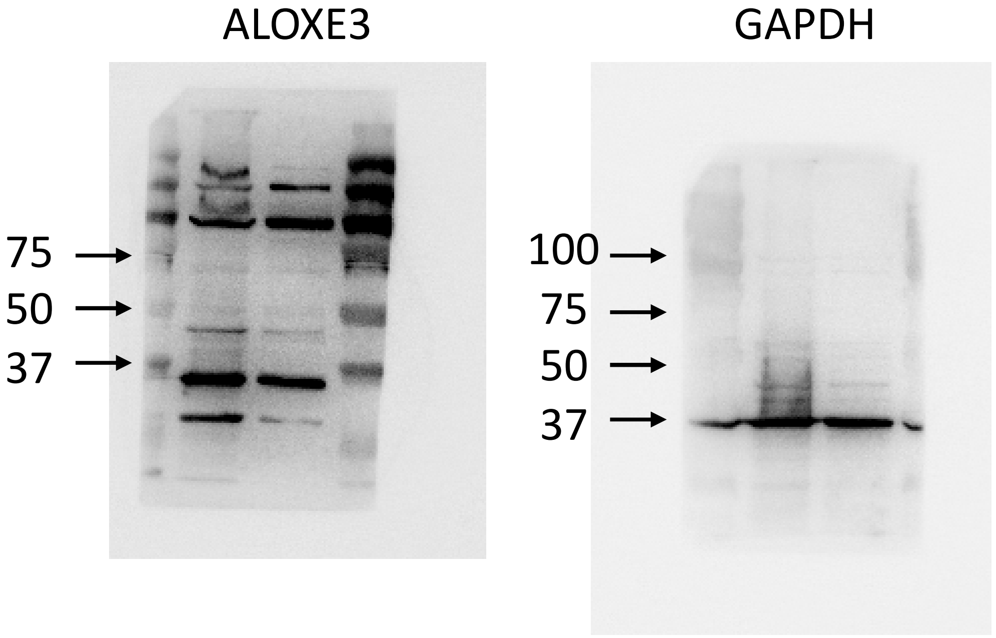
ALOXE3
75
50
37
GAPDH
100
75
50
37

Figure 6O

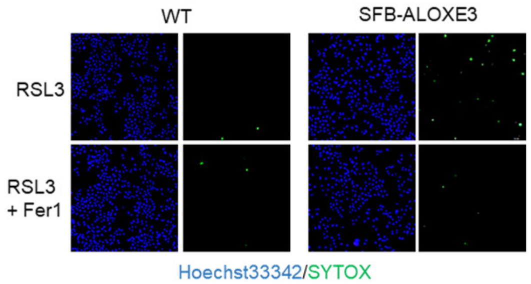

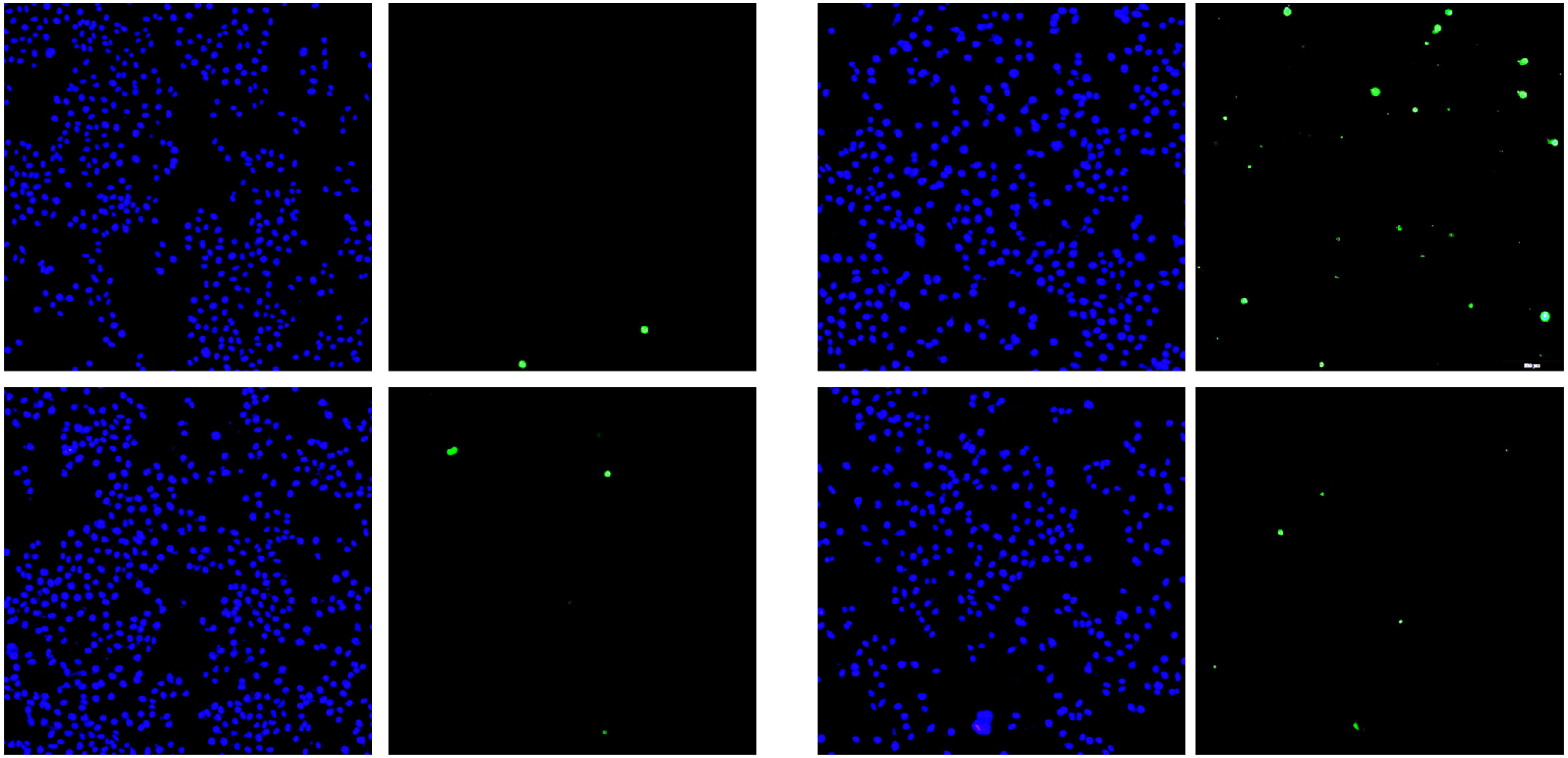

Figure 7B

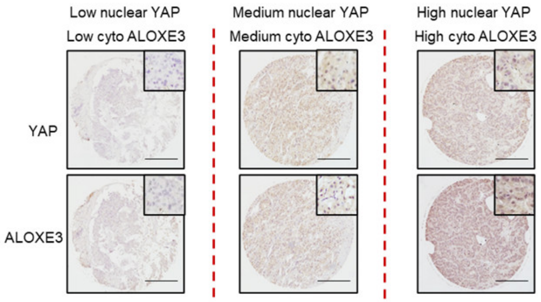
Low nuclear YAP
Low cyto ALOXE3
Medium nuclear YAP
Medium cyto ALOXE3
High nuclear YAP
High cyto ALOXE3
YAP
ALOXE3

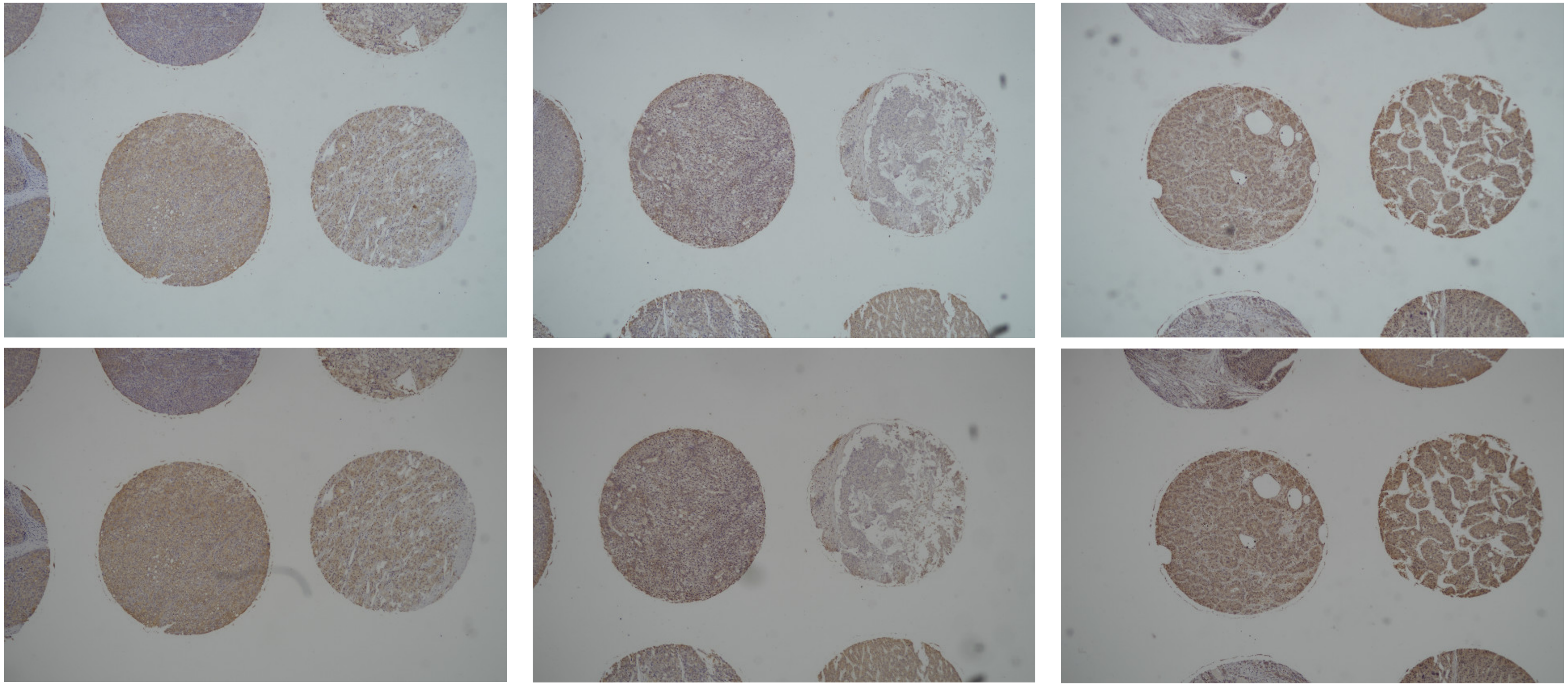

Figure 8C

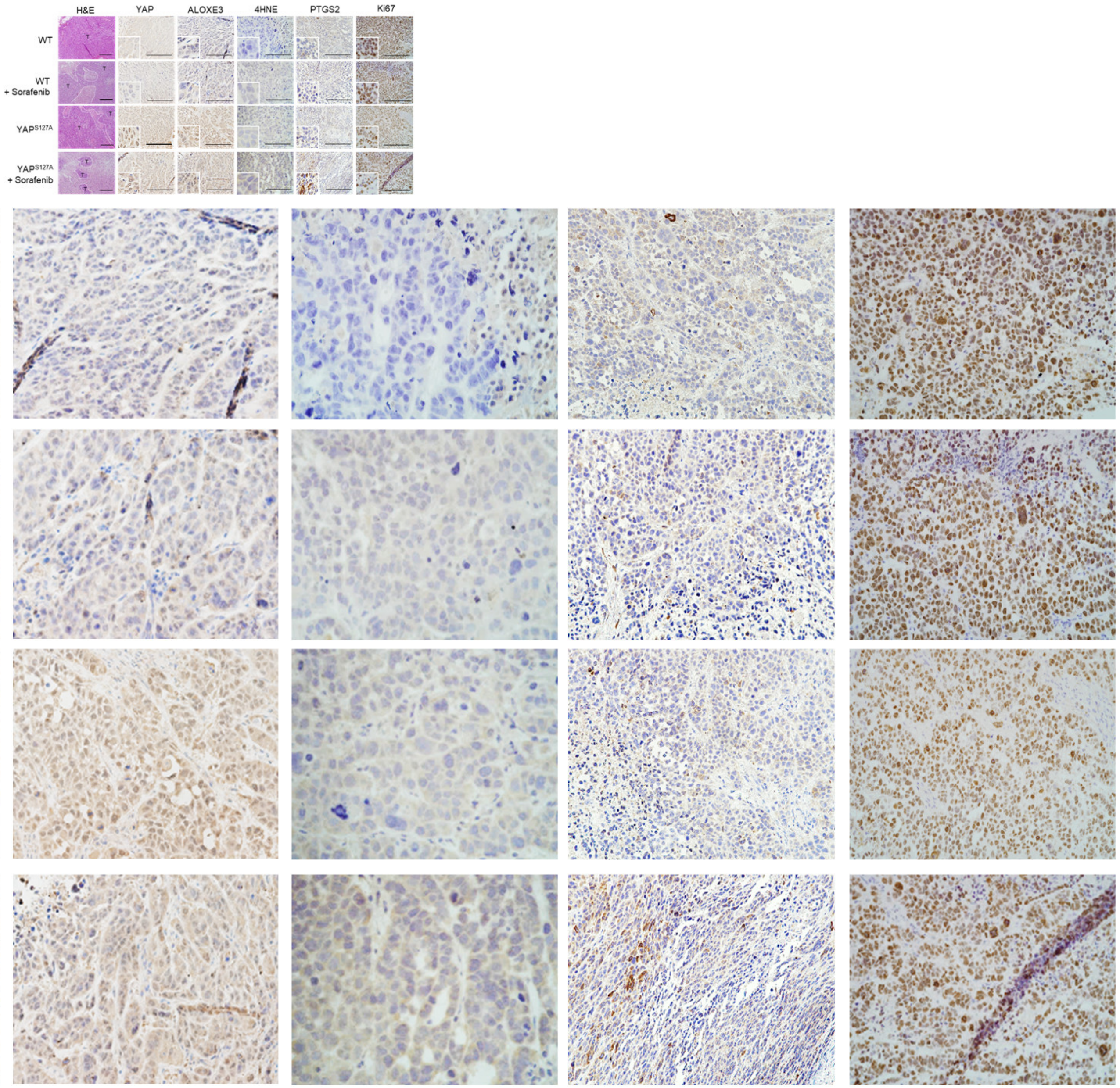
H&E
YAP
ALOXE3
4HNE
PTGS2
Ki67
WT
WT + Sorafenib
YAP^{S127A}
YAP^{S127A} + Sorafenib

Supplementary figure 1A

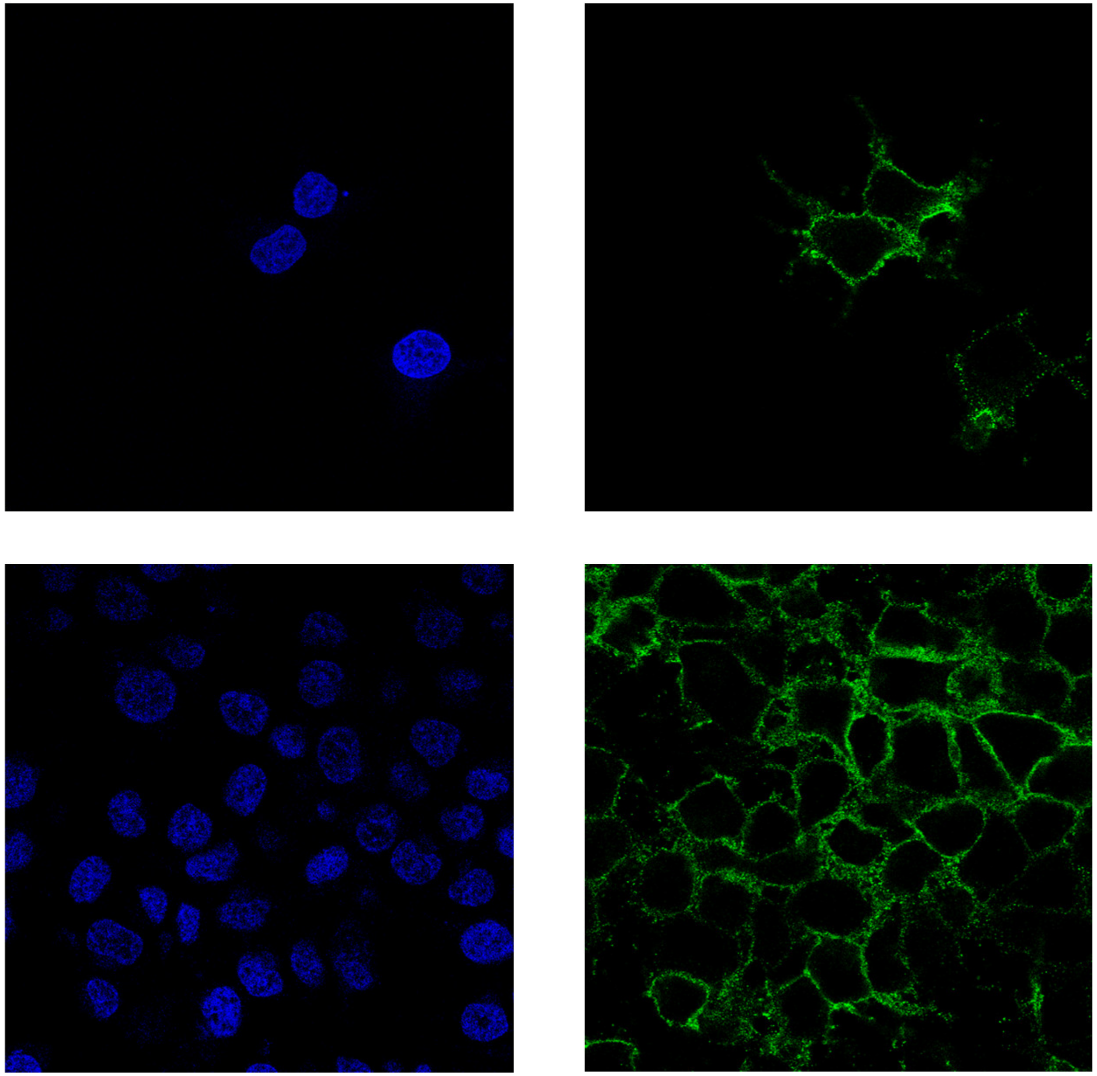

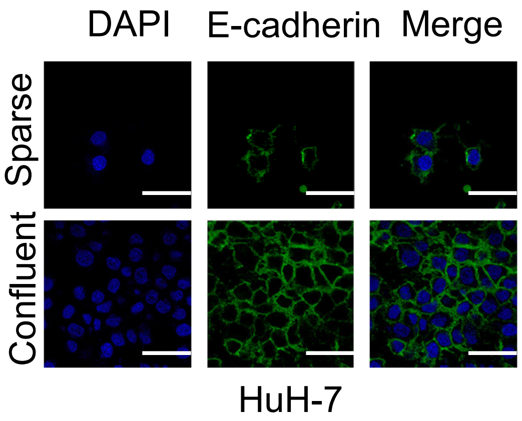

Supplementary figure 1A

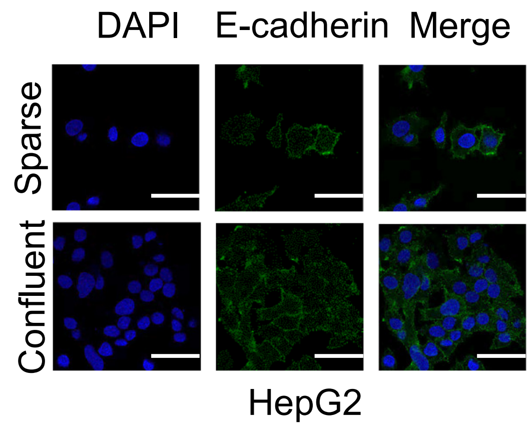

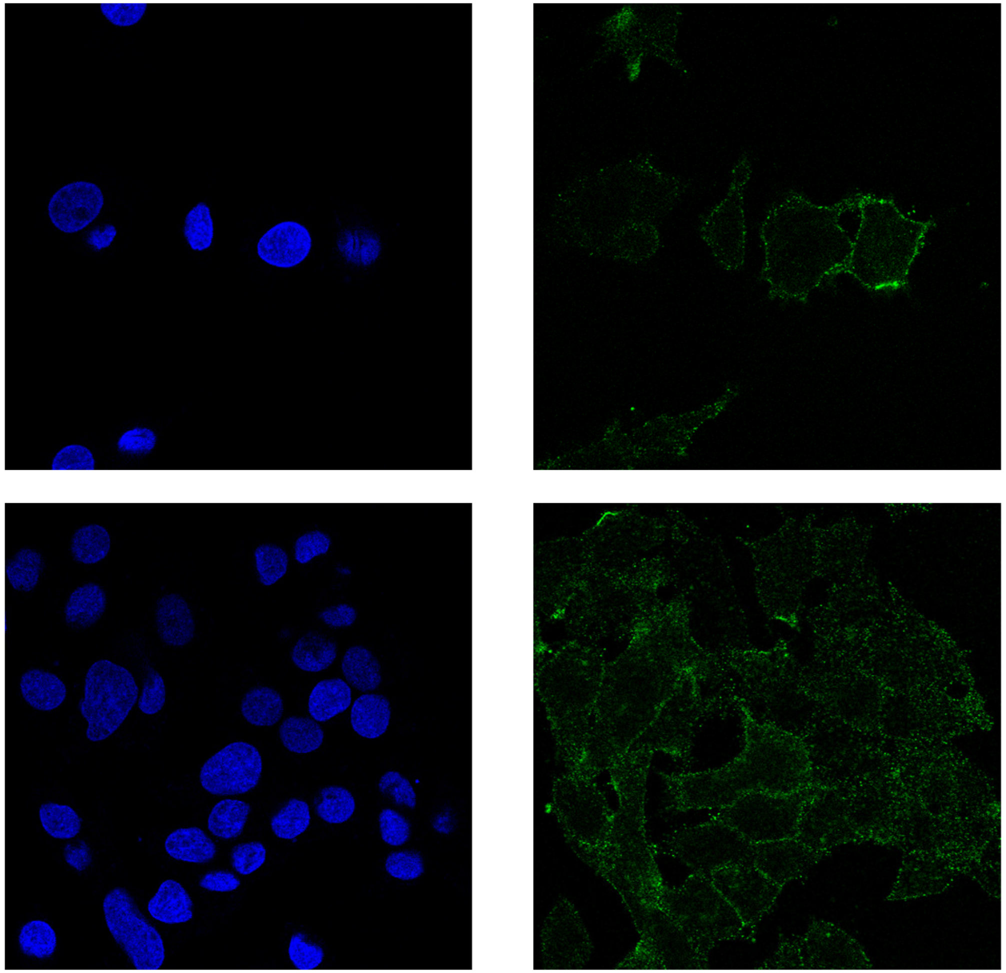

Supplementary figure 1A

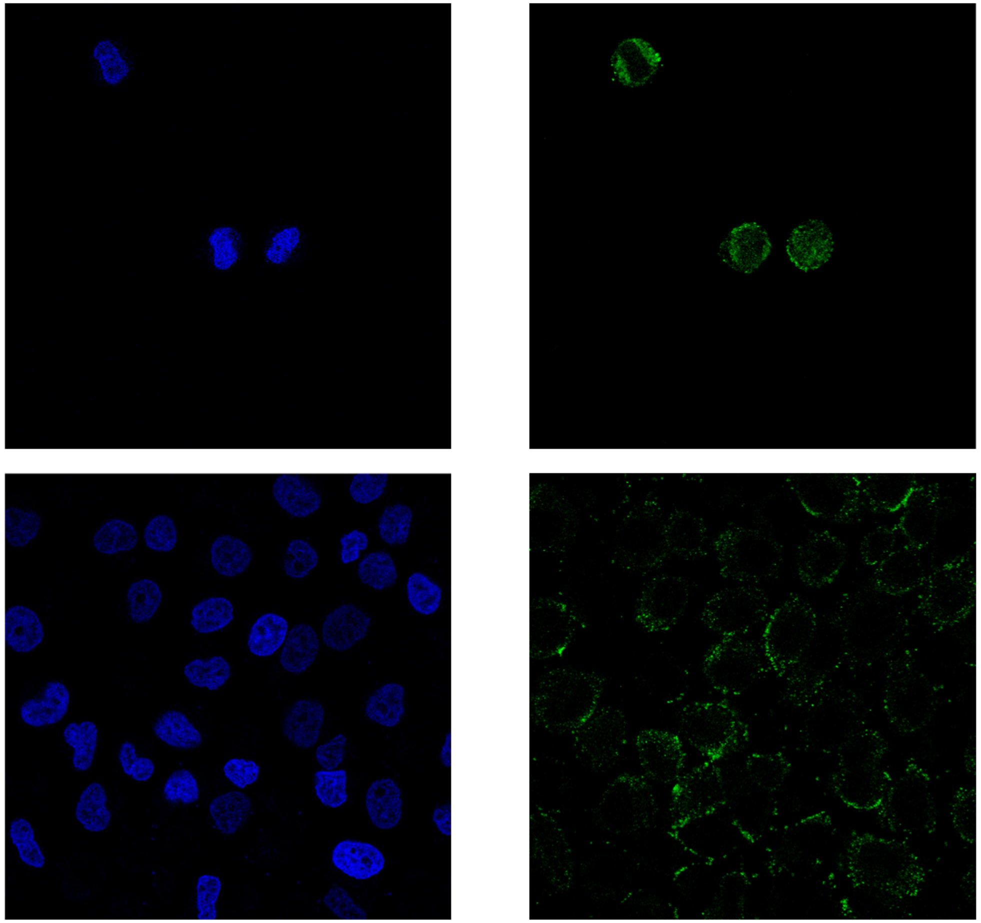

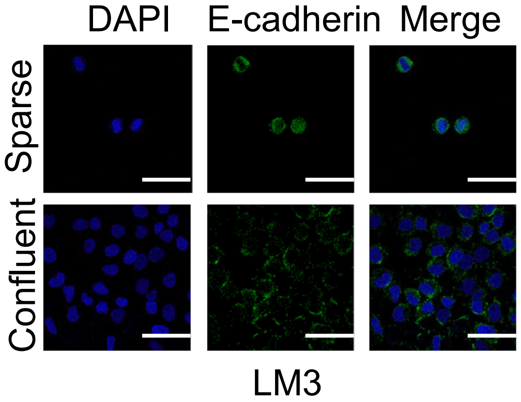

Supplementary figure 1A

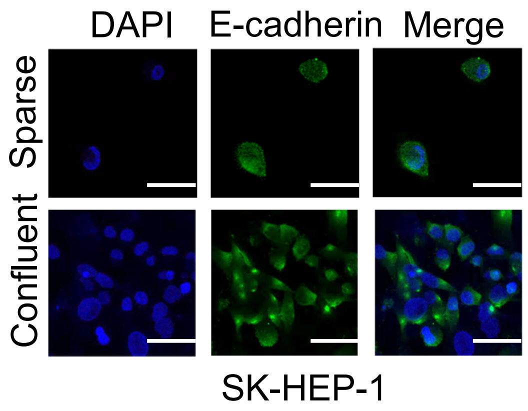

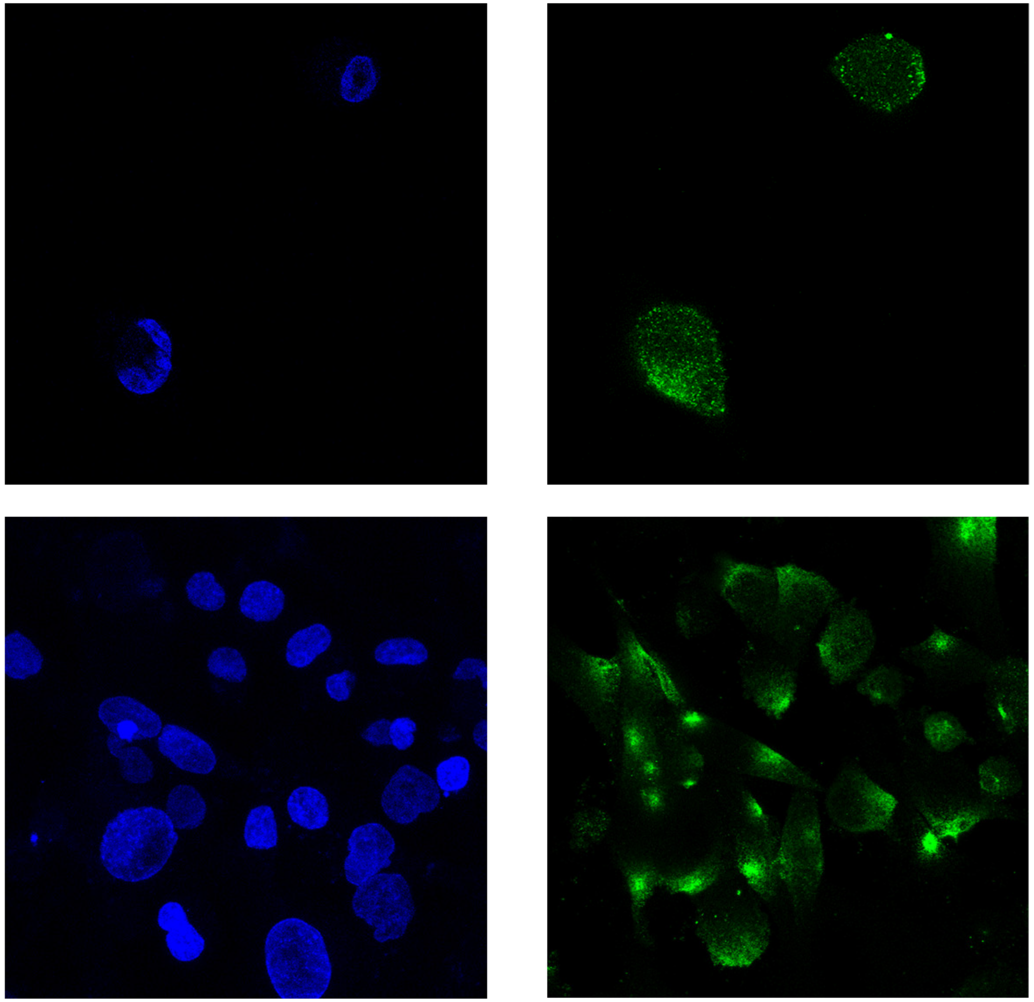

Supplementary figure 1B

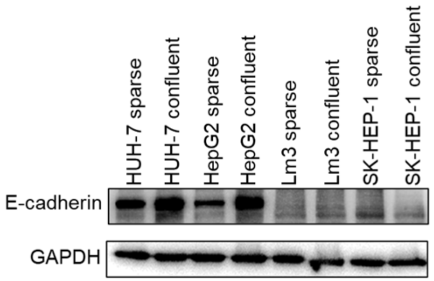

E-cadherin

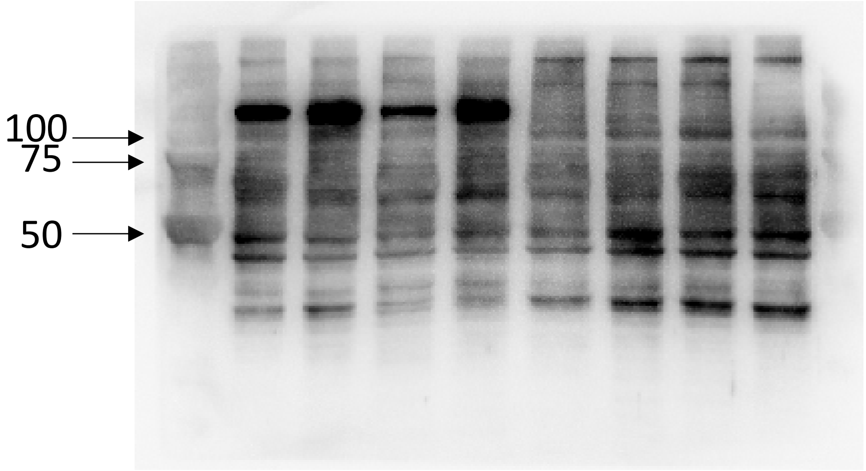

GAPDH

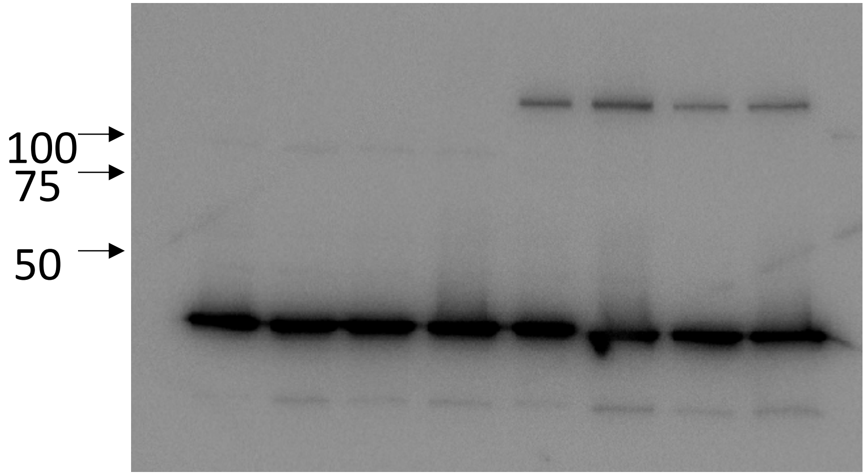

Supplement: Supplementary file 1 [file DataSheet1.zip › raw data/microscope images and western blot full scans.pdf]
