# Supplementary figures and images for: Oncogenic Activation of YAP Signaling Sensitizes Ferroptosis of Hepatocellular Carcinoma via ALOXE3-Mediated Lipid Peroxidation Accumulation
Source: Front Cell Dev Biol. 2021 Dec 16;9:751593. doi: 10.3389/fcell.2021.751593 (PMC8717939; doi:10.3389/fcell.2021.751593)

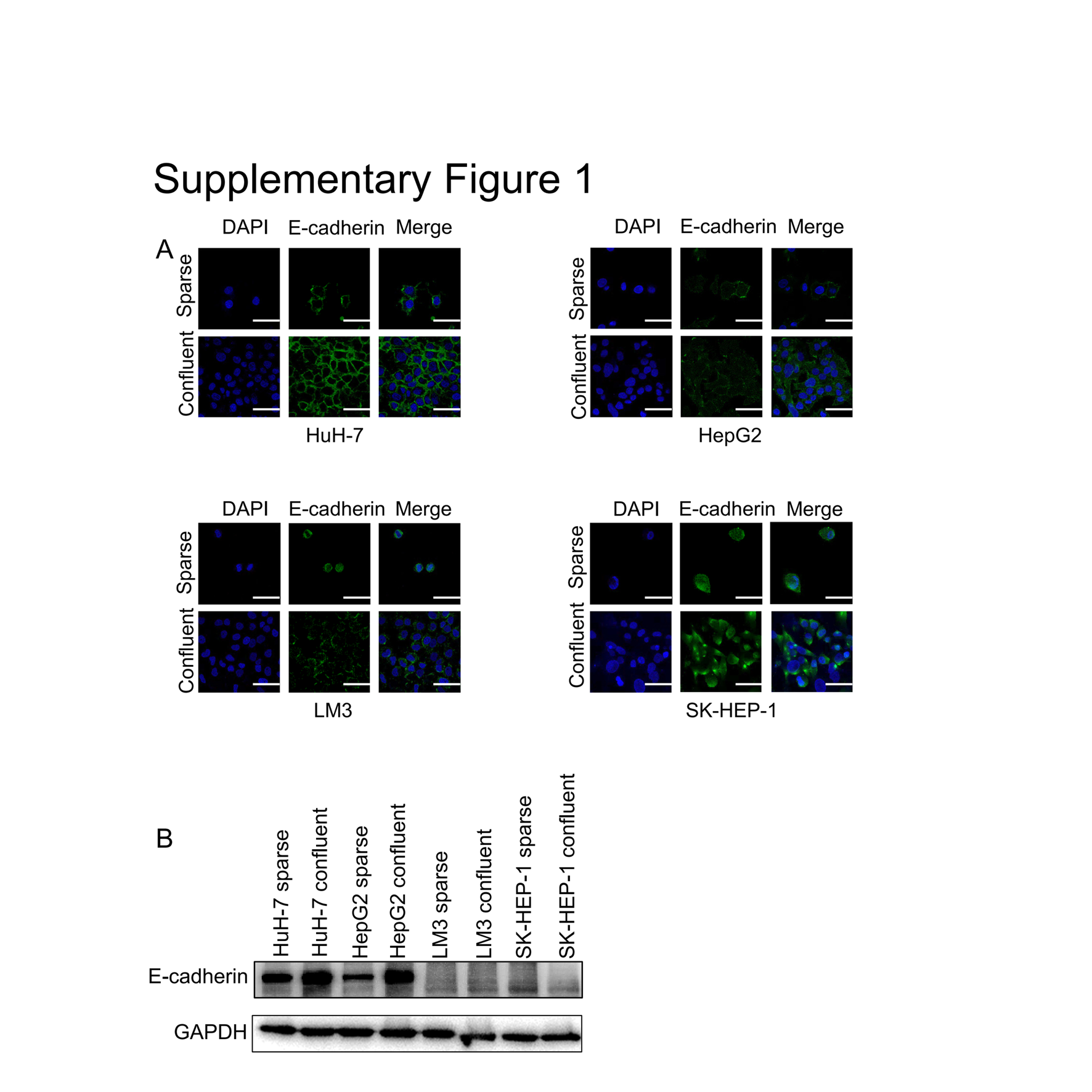

Supplement: Supplementary file 1 [file DataSheet1.zip › raw data/supplementary Figure 1.tif]
